# Brain Volumetric Changes Post-COVID-19: A Systematic Review

**DOI:** 10.3390/brainsci15121255

**Published:** 2025-11-22

**Authors:** Engy Elkoury, Asmaa Yehia, Elisabeth C. Caparelli, Yonas E. Geda, Dakota Ortega, Nelson Yamada, Sasha Hakhu, Scott C. Beeman, Thomas J. Ross, Yihong Yang, Yuxiang Zhou, John D. Port, Osama A. Abulseoud

**Affiliations:** 1 Faculty of Medicine, Tanta University, Tanta 31511, Egypt; 2 Department of Psychiatry and Psychology, Mayo Clinic Arizona, 5777 E Mayo Blvd, Phoenix, AZ 85054, USA; 3 Department of Neuroscience, Graduate School of Biomedical Sciences, Mayo Clinic College of Medicine, Phoenix, AZ 85259, USA; 4 Department of Medical Physiology, Faculty of Medicine, Mansoura University, Mansoura 35516, Egypt; 5 Neuroimaging Research Branch, National Institute on Drug Abuse, National Institutes of Health, Baltimore, MD 21224, USA; 6 Department of Neurology, Franke Global Neuroscience Education Center, Barrow Neurological Institute, Phoenix, AZ 85013, USA; 7 Preclinical Imaging Core, Arizona State University, Tempe, AZ 85257, USA; 8 School of Biological and Health Systems Engineering, Arizona State University, Tempe, AZ 85282, USA; 9 Department of Radiology, Mayo Clinic Arizona, Phoenix, AZ 85259, USA; zhou.yuxiang@mayo.edu; 10 Department of Radiology, Mayo Clinic, Rochester, MN 55905, USA; port.john@mayo.edu; 11 Department of Psychiatry and Psychology, Mayo Clinic, Rochester, MN 55906, USA

**Keywords:** SARS-CoV-2, COVID-19, post-COVID, sMRI, brain volumetric changes

## Abstract

**Background:** The potential long-term effects of Severe Acute Respiratory Syndrome Coronavirus (SARS-CoV-2) infection on the brain structure have not yet been fully elucidated. Even though existing studies have reported structural changes in the post-COVID-19 period, the results remain highly inconsistent and controversial. As such, identifying an imaging biomarker for post-COVID brains is still under investigation. This review aims to comprehensively summarize the structural MRI (sMRI) studies that focus on volumetric brain changes at least two weeks following COVID-19 infection. **Methods:** A systematic literature search was conducted on PubMed, SCOPUS, Web of Science, EMBASE, and Google Scholar up to 9 September 2025. Studies that utilized sMRI to assess volumetric brain changes post-COVID at greater than two weeks following infection were included. Exclusion criteria encompassed research involving pediatric or adolescent populations and imaging modalities other than sMRI. Preprints, reviews, case reports, case series and post-mortem studies were also excluded. **Results:** Forty-one studies satisfied the inclusion criteria and consisted of 2895 patients and 1729 healthy controls. Despite the wide variability in image acquisition protocols, data processing methods, and comorbidities between studies, multiple studies reported statistically significant volumetric reductions in the hippocampus, amygdala, thalamus, basal ganglia, nucleus accumbens and the cerebellum months to years after infection, especially in older hospitalized patients with severe COVID-19. **Conclusions:** The emerging literature reports long-term volume changes across various brain regions in individuals previously infected with COVID-19; however, the evidence is inconsistent. Specific imaging biomarkers following exposure to SARS-CoV-2 infection and the underlying mechanisms of these changes are yet to be identified. Future studies with harmonized imaging protocols, longitudinal designs, and integrated biomarker and clinical data are needed to define robust biomarkers and elucidate the pathophysiology of these findings.

## 1. Introduction

The Coronavirus Disease 2019 (COVID-19) pandemic, caused by Severe Acute Respiratory Syndrome Coronavirus (SARS-CoV-2), has infected hundreds of millions, with an estimated global mortality of over 14 million lives [1]. It is well established that SARS-CoV-2 not only affects the pulmonary system but also the cardiovascular, renal, gastrointestinal, hepatic and hematologic systems [2]. In addition, growing evidence suggests that the central nervous system (CNS) is also vulnerable to the virus’s impact [3,4], with an increasing number of people struggling with persistent neuropsychiatric manifestations [5,6,7,8,9,10,11,12]. These include fatigue, headaches, dyspnea, cognitive impairment, autonomic dysfunction and other neurologic manifestations [13]. This constellation of symptoms has been referred to as Long-COVID or post-COVID syndrome. It has been reported that up to 10% of adults infected with COVID-19 suffer from Long-COVID symptoms [14]. Evidence of SARS-CoV-2 brain involvement has been supported by the presence of viral RNA and proteins in various brain regions [15]. There is emerging evidence that SARS-CoV-2 affects the brain’s grey matter [16], causing structural changes [17]. The reduction in grey matter volume (GMV) could be attributed to neuroinflammation and hypoxia [18,19], and an increase GMV may be due to brain swelling or compensatory mechanisms [20,21].

The long-term effect of this respiratory viral infection on the brain structure has been documented in several imaging studies and recent reviews [22,23,24,25,26,27,28,29]. However, there is a wide variability in the infection severity from asymptomatic to requiring ICU admission and in the post-infection state from complete recovery to post-COVID syndrome with varying presentations at different time points. This variability is evident in the results of imaging studies with reports of volumetric reduction, increase, or no change in many brain regions. As such, identifying an imaging biomarker for post-COVID brain is still under investigation despite the fact that many studies document structural differences between patients and controls years after infection.

Prior systematic reviews investigating brain volumetric alterations following COVID-19 infection have provided relevant findings. However, some included a small number of studies [26,28].The absence of non-COVID-19 control groups in some reviews makes it difficult to attribute observed structural alterations specifically to SARS-CoV-2 infection [29]. Additionally, some studies [26] focused specifically on asymptomatic COVID-19 individuals, thereby excluding symptomatic cases (post-COVID-19 symptoms or neurological sequelae). Given that a significant proportion of COVID-19 survivors report persistent symptoms such as fatigue and cognitive impairment, studying symptomatic populations is crucial for understanding the full neuropsychiatric burden of the disease. Therefore, we incorporated both symptomatic and asymptomatic patients to capture the full spectrum of brain volumetric changes associated with SARS-CoV-2. Furthermore, previous reviews often limited their post-infection timeframes, excluding early recovery phases [26]. In contrast, our review aimed to encompass all stages of recovery—from two weeks post-infection onward—without imposing an upper time limit. This allows for the identification of the potentially progressive nature of post-COVID neurological changes and provides a better understanding of how brain structure may be affected over time.

## 2. Methods

### 2.1. Protocol and Registration

Our systematic review adhered to the principles outlined in the PRISMA 2020 statement guidelines [30] (Supplementary Tables S1 and S2) and was prospectively registered in the Open Science Framework [31] on 25 September 2025 (DOI: 10.17605/OSF.IO/B72QD).

### 2.2. Eligibility Criteria

Eligible studies must have satisfied all the following inclusion criteria: observational studies—prospective, retrospective and cross-sectional studies of adult patients with a previous history of COVID-19 infection including post-COVID disease, post-COVID neurological disease or completely recovered patients; with control groups (negative history of COVID-19) or before–after studies with no control group. Studies must have reported at least one of the following volumetric outcomes: total brain, total white matter, total grey matter, hippocampal, amygdala, thalamic, caudate, putamen, pallidum, nucleus accumbens, or cerebellar volumes. MRI must have been acquired at least 2 weeks following COVID-19 infection. Studies including pediatric or adolescent populations, studies reporting results other than sMRI volumetric changes (e.g., cortical thickness), studies utilizing imaging modalities other than sMRI, and post-mortem studies were excluded. Preprints, reviews, case series, case reports, and conference papers were also excluded from the study.

### 2.3. Search Strategy and the Selection Process

A systematic literature search was conducted on five major databases, including PubMed, SCOPUS, Web of Science, EMBASE, and Google Scholar, up to 9 September 2025. Firstly, a broad search strategy was applied. It included the following terms: ((COVID-19) OR (SARS-CoV-2) OR (COVID-19)) AND (Brain) AND ((Magnetic Resonance Imaging) OR (MRI)). Secondly, we targeted our search on the Cerebellum, Caudate and the Nucleus Accumbens to retrieve specific articles that may have been overlooked. The query used was as follows: ((COVID 19) OR (Corona virus) OR (SARS-CoV-2)) AND ((Nucleus Accumbens) OR (Caudate) OR (cerebell*)) AND ((MRI) OR (Magnetic resonance imaging)) (Supplementary Table S3). The screening and selection processes were done by two independent authors (EE and AY) through the covidence software with no restrictions imposed on time or language. Additionally, the reference list of each identified study was screened for eligibility. The degree of inter-rater agreement was quantified using Cohen’s kappa for study screening. The same two reviewers (EE, AY) independently extracted all relevant information from the included studies and recorded it in Microsoft Excel. The extracted data covered study characteristics (such as study design, sample size, number of participants in each group, and the interval between infection and MRI scan), patient characteristics (including mean age with standard deviation and male-to-female ratio), and the primary outcomes (post-COVID brain volumetric changes across different brain regions and their corresponding *p*-values). Any discrepancies in data extraction were resolved through discussion between the reviewers (EE, AY).

A quantitative meta-analysis was not performed because the included studies showed considerable differences. The main sources of variation were differences in MRI protocols (e.g., field strength and sequences), image processing software (such as FreeSurfer, FSL, or CAT12), patient characteristics (severity of illness and time since infection) and reported outcome measures. A narrative synthesis was therefore used to summarize the findings, highlight patterns and inconsistencies, and suggest directions for future research.

### 2.4. Quality Assessment and Risk of Bias Evaluation

Two independent reviewers (EE, AY) assessed the quality of the included studies using the National Institutes of Health (NIH) National Heart, Lung, and Blood Institute (NHLBI) Quality Assessment Tool for Observational Cohort and Cross-Sectional Studies [32]. Cohen’s kappa was used to evaluate the level of agreement between the two reviewers and any conflicts were resolved through discussion between authors. The overall risk of each study was categorized as *good*, *fair*, or *poor* based on the number of questions answered ‘Yes’ or ‘No.’ Particular emphasis was placed on questions addressing the recruitment of study groups (Q2), the consistency of COVID-19 exposure measures (Q9), and the adequacy of statistical comparisons (Q14), as these were deemed most relevant to the objectives of this review (Supplementary Table S4).

## 3. Results

### 3.1. Literature Search and Study Selection

**Figure 1:**
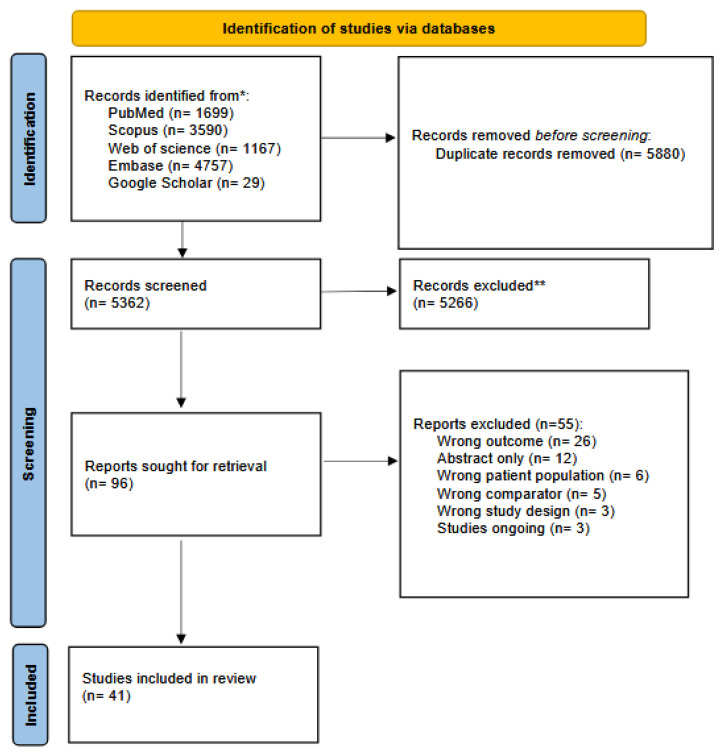
PRISMA flow diagram. * all records identified ** records excluded.

We conducted a comprehensive literature search across five databases and identified 11,242 records. After removing 5880 duplicates, 5362 records remained for title and abstract screening. Of these, 5266 were excluded because they did not meet the pre-specified inclusion criteria. Ninety-six full-text reports were retrieved and screened in the full-text screening phase. The agreement between reviewers was a Cohen’s κ of 0.84 at this stage (almost perfect agreement). The full-text articles were assessed for eligibility, and 55 were excluded at this stage. Ultimately, 41 studies with a total of 2895 patients and 1729 controls satisfied all inclusion criteria and were incorporated into our review [17,18,20,33,34,35,36,37,38,39,40,41,42,43,44,45,46,47,48,49,50,51,52,53,54,55,56,57,58,59,60,61,62,63,64,65,66,67,68,69] (Figure 1).

### 3.2. Characteristics of the Included Studies

In total, 41 studies comprising 4624 adult participants were included in this review. Females were more represented than males in both the COVID-19 and control groups, with 1610 females vs. 1273 males in the COVID-19 groups and 993 females vs. 729 males in the control groups. Participants ranged in age from 20 to 71 years. Geographically, the studies were conducted across a wide range of countries: nine in China [18,20,43,49,53,57,58,59], seven in Italy [35,37,38,45,48,52,62], five in Germany [33,34,42,61,68], four in the UK [17,41,44,64], three in Russia [47,55,60], three in Spain [39,40,66], two in Turkey [46,56], and one each in Austria [67], Australia [69], Argentina [36], Poland [50], Brazil [65], the USA [54], Hungary, and India. Detailed characteristics of each study are provided in (Table 1, Table 2, Table 3, Table 4, Table 5, Table 6, Table 7, Table 8 and Table 9). Most of the included studies focused on the hippocampus (n = 24), amygdala (n = 17), thalamus (n = 15) and total brain volume (n = 13). All studies used T1 MRI scans to examine the volumetric changes post-COVID; however, a wide variety of techniques were applied. (Details on MRI scanning and image processing can be found in Supplementary Table S5).

### 3.3. Risk of Bias and Quality Assessment

The quality of the included studies varied, ranging from good to poor. Twenty-five studies were rated as good quality [17,18,34,35,36,37,38,39,40,42,43,44,45,48,49,51,54,56,59,61,64,65,66,67,68], eleven as fair [20,38,41,43,46,52,55,58,62,63,69], and five as poor [33,47,53,57,60]. The Cohen’s κ was 0.60, indicating moderate agreement. The most common reasons for lower quality ratings were insufficient statistical adjustment for key covariates (Q14), limited or unclear information on participant recruitment (Q2), and the use of undefined or inconsistent measures of COVID-19 exposure (Q9) (Supplementary Table S4; Supplementary Figure S1).

### 3.4. Total Brain Volume

The majority of studies (10 out of 13, 77%; n = 789 patients, n = 323 controls) found no significant difference in total brain volume [35,36,37,38,45,46,49,51,54,60], while three studies (23%; n = 759 patients, n = 492 controls) reported significantly lower volumes [17,33,41] (Table 1). Specifically, the severe COVID-19 group in Bendella et al. [33] had a mean volume 53.48 mL lower than controls, and the C-MORE study [41] reported a mean volume 35 mL lower in its COVID-19 group compared to controls.

**Table 1:** Total brain volume.

First Author, Year Country	Study Design	Sample Characteristics		COVID-19 Severity	Time Between COVID-19 and MRI	Direction	*p*
		COVID-19	Control				
Douaud et al., 2022 [17] UK	Longitudinal Pre- and post-infection vs. controls	n = 401 62.1 ± 6.7 years (m:f) (172:229)	n = 384 63.3 ± 7.1 years (m:f) (164:220)	Recovered	141 ± 79 days (second scan)	↓	<0.05
Bendella et al., 2023 [33] Germany	Prospective	n = 99 (n = 51 mild; n = 48 severe) Mild: 45.7 ± 12.4 years; Severe: 50.6 ± 12.0 years Mild (m:f) (28:28), Severe (m:f) (25:23)	n = 56 47.0 ± 13.3 years (m:f) (26:25)	Recovered	Mild: 8.7 ± 4.8 months Severe: 10.7 ± 5 months	↓	0.003
C-MORE 2023 [41] UK	Prospective	n = 259 57.0 ± 12.2 years (m:f) (158:101)	n = 52 49.3 ± 13.9 years (m:f) (30:22)	Recovered	5 (4.2–6.3) months median (IQR)	↓	0.006
Cataldo et al., 2024 [36] Argentina	Cross-sectional	n = 109 48.4 ± 8.0 years (m:f) (30:79)	n = 28 45.2 ± 9.9 years (m:f) (9:19)	Long COVID	2 years	↔	0.06
Cecchetti et al., 2022 [38] Italy	Cross-sectional	n = 36 58.5 ± 13.3 years (m:f) (25:11)	n = 36 56.9 ± 13.6 years (m:f) (20:16)	Recovered	2 months	↔	0.1
Perlaki et al., 2024 [51] Hungary	Cross-sectional	n = 38 26.6 ± 5 years (m:f) (14:24)	n = 37 25.9 ± 2.8 years (m:f) (14:23)	Recovered	Median (IQR): 178 (112–241.3) days	↔	0.451
Rothstein et al., 2023 [54] USA	Retrospective	n = 24 46.9 years (range 22–60 years) (m:f) (5: 19)	Sex and age matched healthy controls	Post-COVID syndrome	85 days	↔	1.0
Capelli et al., 2024 [35] Italy	Retrospective	n = 145 (n = 61 COVID-CD; n = 48 COVID-OD) COVID-CD median (IQR) 57 (50–63) years; COVID-OD median (IQR) 49 (35–57) years COVID-CD (m:f) (23:38), COVID-OD (m:f) (34:50)	n = 17 Median (IQR) 51 (41–52) years (m:f) (10:7)	COVID-19-related cognitive and olfactory dysfunction	COVID-CD median (IQR) 210 (53–446) days COVID-OD median (IQR) 237 (180–323) days	↔	>0.05
Cattarinussi et al., 2022 [37] Italy	Cross-sectional	n = 79 42.8 ± 13.8 years (m:f) (33:46)	n = 17 35.8 ± 11.7 years (m:f) (11:6)	Recovered	132 ± 67 days	↔	0.307
Invernizzi et al., 2024 [45] Italy	Case–control Longitudinal	n = 13 23.76 ± 2.82 years (m:f) (6:7)	n = 27 24.1 ± 2.3 years (m:f) (8:19)	Recovered	1–11 months	↔	0.311
Kamasak et al., 2023 [46] Turkey	Cross-sectional	n = 50 38.10 ± 5.85 years (m:f) (25:25)	n = 50 38.78 ± 6.16 years (m:f) (25:25)	Recovered	17 days	↔	0.182
Trufanov et al., 2025 [60] Russia	Cross-sectional Case–control	n = 24 49.16 ± 10.65 years (m:f) (12:11)	n = 20 42.84 ± 8.93 years (m:f) (6:12)	Post-COVID-syndrome	4–6 months	↔	0.587
Niu et al., 2025 [49] China	Prospective Longitudinal	n = 271 40.16 ± 10.21 years (m:f) (121:150)	n = 67 37.76 ± 11.64 years (m:f) (28:39)	Recovered	1 month and 3 months	↔	0.96

Abbreviations: m:f, male: female; IQR, interquartile range; CD, cognitive dysfunction; OD, olfactory dysfunction; TIV, total intracranial volume; NR, not reported.

### 3.5. Total Grey Matter Volume

Findings for total grey matter volume were heterogeneous. Six studies (67%; n = 795 patients, n = 246 controls) reported no significant differences [35,36,37,38,49,52,64,67], while three studies by Bendella et al. [33], C-MORE [41] and Kamasak et al. [46] (25%, n = 408 patients, n = 158 controls) reported lower volumes. Only Lu et al. (8%; n = 60 patients, n = 39 controls) reported higher volumes in patients compared to controls [20] (Table 2). Among studies reporting significant reductions in grey matter volume, Bendella et al. [33] observed a 39.49 mL decrease in severe patients compared to controls, consistent with the C-MORE study [41], which reported a 32.6 mL reduction, and Kamasak et al. [46], who found a 38.66 mm^3^ decrease.

**Table 2:** Total grey matter volume.

First Author, Year	Study Design	Sample Characteristics		COVID-19 Severity	Time Between COVID-19 and MRI	Direction	*p*
		COVID-19	Control				
Bendella et al., 2023 [33] Germany	Prospective	n = 99 (n = 51 mild; n = 48 severe) Mild: 45.7 ± 12.4 years; Severe: 50.6 ± 12.0 years Mild (m:f) (28:28), Severe (m:f) (25:23)	n = 56 47.0 ± 13.3 years (m:f) (26:25)	Recovered	Mild: 8.7 ± 4.8 months Severe: 10.7 ± 5 months	↓	<0.001
C-MORE 2023 [41] UK	Prospective	n = 259 57.0 ± 12.2 years (m:f) (158:101)	n = 52 49.3 ± 13.9 years (m:f) (30:22)	Recovered	5 (4.2–6.3) months median (IQR)	↓	<0.001
Kamasak et al., 2023 [46] Turkey	Cross-sectional	n = 50 38.10 ± 5.85 years (m:f) (25:25)	n = 50 38.78 ± 6.16 years (m:f) (25:25)	Recovered	17 days	↓	<0.05
Lu et al., 2020 [20] China	Prospective	n = 60 44.1 ± 16.0 years (m:f) (34:26)	n = 39 45.9 ± 13.9 years (m:f) (22:17)	Recovered	97.5 ± 8.0 days	↑	0.02
Cataldo et al., 2024 [36] Argentina	Cross-sectional	n = 109 48.4 ± 8.0 years (m:f) (30:79)	n = 28 45.2 ± 9.9 years (m:f) (9:19	Long COVID	2 years	↔	0.09
Cecchetti et al., 2022 [38] Italy	Cross-sectional	n = 36 58.5 ± 13.3 years (m:f) (25:11)	n = 36 56.9 ± 13.6 years (m:f) (20:16)	Recovered	2 months	↔	0.15
Cattarinussi et al., 2022 [37] Italy	Cross-sectional	n = 79 42.8 ± 13.8 years (m:f) (33:46)	n = 17 35.8 ± 11.7 years (m:f) 11:6	Recovered	132 ± 67 days	↔	0.227
Capelli et al., 2024 [35] Italy	Retrospective	n = 145 (n = 61 COVID-CD; n = 48 COVID-OD) COVID-CD median (IQR) 57 (50–63) years; COVID-OD median (IQR) 49 (35–57) years COVID-CD (m:f) (23:38), COVID-OD (m:f) (34:50)	n = 17 Median (IQR) 51 (41–52) years (m:f) (10:7)	COVID-19-related cognitive and olfactory dysfunction	COVID-CD median (IQR) 210 (53–446) days COVID-OD median (IQR) 237 (180–323) days	↔	>0.05
Niu et al., 2025 [49] China	Prospective Longitudinal	n = 271 40.16 ± 10.21 years (m:f) (121:150)	n = 67 37.76 ± 11.64 years (m:f) (28:39)	Recovered	1 month and 3 months	↔	0.96
Pelizzari et al., 2022 [52] Italy	Cross-sectional	n = 22 median (IQR) 45.7 (34.8–53.4) years (m:f) (9:13)	n = 21 median (IQR) 37.6 (28.4–56.6) years (m:f) (6:15)	Recovered	7.3 (3.2) Months median (IQR)	↔	NR
Gupta et al., 2024 [64] UK	Cross-sectional	n = 21 median (IQR) 54(49–61) years (m:f) (15:6)	n = 10 median (IQR) 58(52–67) years (m:f) (7:3)	Post-COVID	5–7 months	↔	1.0
Haider et al., 2025 [67] Austria	Prospective cohort	n = 112 45 ± 15 years (m:f) (42:70)	n = 50 44 ± 19 years (m:f) (22:28)	Post-COVID	228 ± 140 days	↔	0.390

Abbreviations: m:f, male: female; IQR, interquartile range; CD, cognitive dysfunction; OD, olfactory dysfunction; GM, grey matter; TIV, total intracranial volume.

### 3.6. Total White Matter Volume

Six studies (n = 824 patients, n = 289 controls) examined the total white matter volume changes, and none showed statistically significant differences [33,36,38,41,46,49] (Table 3).

**Table 3:** Total white matter volume.

First Author, Year Country	Study Design	Sample Characteristics		COVID-19 Severity	Time Between COVID-19 and MRI	Direction	*p*
		COVID-19	Control				
Bendella et al., 2023 [33] Germany	Prospective	n = 99 (n = 51 mild; n = 48 severe) Mild: 45.7 ± 12.4 years; Severe: 50.6 ± 12.0 years Mild (m:f) (28:28), Severe (m:f) (25:23)	n = 56 47.0 ± 13.3years (m:f) (26:25)	Recovered	Mild: 8.7 ± 4.8 months Severe: 10.7 ± 5 months	↔	0.32
Cataldo et al., 2024 [36] Argentina	Cross-sectional	n = 109 48.4 ± 8.0 years (m:f) (30:79)	n = 28 45.2 ± 9.9 years (m:f) (9:19)	Long COVID	2 years	↔	0.12
Cecchetti et al., 2022 [38] Italy	Cross-sectional	n = 36 58.5 ± 13.3 years (m:f) (25:11)	n = 36 56.9 ± 13.6 years (m:f) (20:16)	Recovered	2 months	↔	0.13
Niu et al., 2025 [49] China	Prospective Longitudinal	n = 271 40.16 ± 10.21 years (m:f) (121:150)	n = 67 37.76 ± 11.64 years (m:f) (28:39)	Recovered	1 month and 3 months	↔	0.96
C-MORE 2023 [41] UK	Prospective	n = 259 57.0 ± 12.2 years (m:f) (158:101)	n = 52 49.3 ± 13.9 years (m:f) (30:22)	Recovered	5 (4.2–6.3) months median (IQR)	↔	0.661
Kamasak et al., 2023 [46] Turkey	Cross-sectional	n = 50 38.10 ± 5.85 years (m:f) (25:25)	n = 50 38.78 ± 6.16 years (m:f) (25:25)	Recovered	17 days	↔	0.505

Abbreviations: m:f, male: female; IQR, interquartile range.

### 3.7. Hippocampal Volume

Hippocampal volume findings were the most varied across the literature. Of 24 studies, 13 (54%; n = 1343 patients, n = 715 controls) reported lower volumes [17,35,39,40,41,43,44,45,46,48,50,59,62]. Among these, studies providing quantitative volumetric data revealed substantial variation in the magnitude of hippocampal volume loss, with reductions relative to controls ranging from approximately 4% to 20% [35,39,44,62]. In contrast, six (25%, n = 222 patients, n = 175 controls) reported higher volumes [20,34,54,57,63,69], and five (21%, n = 343 patients, n = 176 controls) found no significant difference [33,37,56,65,68] (Table 4).

**Table 4:** Hippocampal volume.

First Author, Year Country	Study Design	Sample Characteristics		COVID-19 Severity	Time Between COVID-19 and MRI	Direction	*p*
		COVID-19	Control				
Griffanti et al., 2021 [44] UK	Prospective	n = 51 54.8 ± 13.4 years (m:f) (29:22)	n = 25 52.4 ± 12.8 years (m:f) (15:10)	Recovered	Median (IQR) 2.3 (2.06–2.53) months	↓	0.018
Okrzeja et al., 2024 [50] Poland	Cross-sectional	n = 23 53.52 years (m:f) (6:17)	n = 20 52.15 years (m:f) (7:13)	Post-COVID	6 months	↓	<0.05
Diez-Cirarda et al., 2025 [40] Spain	Cross-sectional	n = 129 49.35 ± 10.29 years (m:f) (34:95)	n = 36 56.9 ± 13.6 years (m:f) (20:16)	Long COVID	14.79 ± 7.17 months	↓	<0.05
Capelli et al., 2024 [35] Italy	Retrospective	n = 145 (n = 61 COVID-CD; n = 48 COVID-OD) COVID-CD median (IQR) 57 (50–63) years; COVID-OD median (IQR) 49 (35–57) years COVID-CD (m:f) (23:38), COVID-OD (m:f) (34:50)	n = 17 Median (IQR) 51 (41–52) years (m:f) (10:7)	COVID-19-related cognitive and olfactory dysfunction	COVID-CD median (IQR) 210 (53–446) days COVID-OD median (IQR) 237 (180–323) days	↓	<0.0001
C-MORE 2023 [41] UK	Prospective	n = 259 57.0 ± 12.2 years (m:f) (158:101)	n = 52 49.3 ± 13.9 years (m:f) (30:22)	Recovered	5 (4.2–6.3) months median (IQR)	↓	<0.001
Kamasak et al., 2023 [46] Turkey	Cross-sectional	n = 50 38.10 ± 5.85 years (m:f) (25:25)	n = 50 38.78 ± 6.16 years (m:f) (25:25)	Recovered	17 days	↓	<0.001
Douaud et al., 2022 [17] UK	Longitudinal Pre- and post-infection vs. controls	n = 401 62.1 ± 6.7 years (m:f) (172:229)	n = 384 63.3 ± 7.1 years (m:f) (164:220)	Recovered	141 ± 79 days (second scan)	↓	<0.05
Zhou et al., 2025 [59] China	Longitudinal	n = 53 (n = 26 SD; n = 27 NSD) SD: 51.5 ± 13.57 years; NSD: 47.33 ± 15.98 years SD (m:f) (18:8), NSD (m:f) (18:9)	n = 31 49.19 ± 17.51 years (m:f) (22:9)	Post-COVID sleep disturbances	3 months	↓	<0.001
Invernizzi et al., 2024 [45] Italy	Case–control Longitu dinal	n = 13 23.76 ± 2.82 years (m:f) (6:7)	n = 27 24.1 ± 2.3 years (m:f) (8:19)	Recovered	1–11 months	↓	0.034
Du et al., 2023 [43] China	Prospective	n = 61 37 ± 14 years (m:f) (61:0)	Patients acted as their own controls	Recovered	21.6 ± 5.2 days	↓	0.04
Muccioli et al., 2023 [48] Italy	Prospective	n = 23 37 ± 14 years (m:f) (11:12)	n = 26 38.5 ± 13.7 years (m:f) (13:13)	Post-COVID olfactory dysfunction	11 ± 5 months	↓	L: 0.003 R: 0.002
Díez-Cirarda et al., 2023 [39] Spain	Cross-sectional	n = 84 50.89 ± 11.25 years (m:f) (26:58)	n = 33 37 ± 14 years (m:f) (13:20)	Post-COVID syndrome	11.08 ± 4.47 months	↓	<0.001
Arrigoni et al., 2024 [62] Italy	Retrospective	n = 51 (n = 16 COVID-CM; n = 35 COVID-OD) COVID-CM median (IQR) 56 (51–61) years; COVID-OD median (IQR): 40 (31–53) years COVID-CM (m:f) (5: 11), COVID-OD (m:f) (10:25)	n = 14 median (IQR) 62 (45–70) years (m:f) (8:6)	Post- COVID olfactory and cognitive impairment	Median (IQR): 264 (208–313) days	↓	L COVID-CM vs. Control: 0.002 R COVID-CM vs. Control: 0.009 BIL COVID-OD vs. Control: 0.001
Lu et al., 2020 [20] China	Prospective	n = 60 44.1 ± 16.0 years (m:f) (34:26)	n = 39 45.9 ± 13.9 years (m:f) (22:17)	Recovered	97.5 ± 8.0 days	↑	L: <0.001 R: 0.013
Besteher et al., 2022 [34] Germany	Cross-sectional	n = 30 47.5 ± 11.5 years (m:f) (13:17)	n = 20 42.95 ± 13.41 years (m:f) (10:10)	Long COVID	8.65 months	↑	L: 0.028 R: 0.046
Hafiz et al., 2022 [63] India	Cross-sectional	n = 46 34.63 ± 11.54 years (m:f) (31:15)	n = 30 33.5 ± 9.74 years (m:f) (23:7)	Recovered	2 weeks	↑	<0.05
Tu et al., 2021 [57] China	Prospective	n = 47 51.8 ± 11.3 years (m:f) (14:33)	n = 47 51.8 ± 11.3 years (m:f) (15:32)	Recovered	6 months	↑	NR
Rothstein et al., 2023 [54] USA	Retrospective	n = 24 46.9 years (range 22–60 years) (m:f) (5:19)	Sex and age matched healthy controls	Post-COVID syndrome	85 days	↑	0.0169
Thapaliya et al., 2025 [69] Australia	Cross-sectional	n = 15 51.65 ± 11.26 years (m:f) (4:11)	n = 15 38.26 ± 12.74 years (m:f) (5:10)	Long COVID	0.60 ± 0.46 years	↑	< 0.01
Bendella et al., 2023 [33] Germany	Prospective	n = 99 (n = 51 mild; n = 48 severe) Mild: 45.7 ± 12.4 years; Severe: 50.6 ± 12.0 years Mild (m:f) (28:28), Severe (m:f) (25:23)	n = 56 47.0 ± 13.3 years (m:f) (26:25)	Recovered	Mild: 8.7 ± 4.8 months Severe: 10.7 ± 5 months	↔	R:0.43 L: 0.57
Taskiran-Sag et al., 2023 [56] Turkey	Cross-sectional case–control	n = 20 35.5 ± 9.5 years (m:f) (10:10)	n = 20 36.3 ± 6.7 years (m:f) (9:11)	Recovered	107 days	↔	NR
Cattarinussi et al., 2022 [37] Italy	Cross-sectional	n = 79 42.8 ± 13.8 years (m:f) (33:46)	n = 17 35.8 ± 11.7 years (m:f) (11:6)	Recovered	132 ± 67 days	↔	0.307
Bispo et al., 2022 [65] Brazil	Cross-sectional	n = 56 37.2 ± 9.4 years (m:f) (20:36)	n = 37 40.2 ± 11.8 years (m:f) (15:22)	Post-COVID symptoms	93.3 ± 26.4	↔	>0.120
Hosp et al., 2024 [68] Germany	Cross-sectional	n = 89 median (IQR) 49 (23) years (m:f) (34:55)	n = 46 median (IQR) 44 (31) years (m:f) (23:23)	Post-COVID	254 (209) days median (IQR)	↔	1.000

Abbreviations: m:f, male: female; IQR, interquartile range; CD, cognitive dysfunction; OD, olfactory dysfunction; SD, sleep disturbances; NSD, none sleep disturbances; TIV, total intracranial volume; R, right; L, left; BIL, bilateral; PCS, post-COVID syndrome; CM, cognitive impairment; NR, not reported.

### 3.8. Amygdala Volume

Lower volume was the most prevalent finding (41%, n = 982 patients, n = 697 controls) [17,35,41,42,48,55,62]. Six studies (35%, n = 291 patients, n = 200 controls) found no significant difference [20,45,56,59,65,68], and four studies by Besteher et al. [34], Hafiz et al. [63], Tu et al. [57] and Lukina et al. [47] (24%, n = 157 patients, n = 127 controls) reported higher volumes (Table 5).

**Table 5:** Amygdala volume.

First Author, Year Country	Study Design	Sample Characteristics		COVID-19 Severity	Time Between COVID-19 and MRI	Direction	*p*
		COVID-19	Control				
Syunyakov et al., 2022 [55] Russia	Longitudinal	n = 24 median (IQR) 71 (68.4–77.0) (m:f) (3:21)	n = 183 median (IQR) 71 (68.4–77.0) (m:f) (33:150)	Recovered	NR	↓	0.044
Douaud et al., 2022 [17] UK	Longitudinal Pre- and post-infection vs. controls	n = 401 62.1 ± 6.7 years (m:f) (172:229)	n = 384 63.3 ± 7.1 years (m:f) (164:220)	Recovered	141 ± 79 days (second scan).	↓	<0.05
Capelli et al., 2024 [35] Italy	Retrospective	n = 145 (n = 61 COVID-CD; n = 48 COVID-OD) COVID-CD median (IQR) 57 (50–63) years; COVID-OD median (IQR) 49 (35–57) years COVID-CD (m:f) (23:38), COVID-OD (m:f) (34:50)	n = 17 Median (IQR) 51 (41–52) years (m:f) (10:7)	COVID-19-related cognitive and olfactory dysfunction	COVID-CD median (IQR) 210 (53–446) days COVID-OD median (IQR) 237 (180–323) days	↓	<0.0001
C-MORE 2023 [41] UK	Prospective	n = 259 57.0 ± 12.2 years (m:f) (158:101)	n = 52 49.3 ±13.9 years (m:f) (30:22)	Recovered	Median (IQR): 5 (4·2–6·3)	↓	< 0.001
Muccioli et al., 2023 [48] Italy	Prospective	n = 23 37 ± 14 years (m:f) (11:12)	n = 26 38.5 ± 13.7 years (m:f) (13:13)	Post-COVID olfactory dysfunction	11 ± 5 months	↓	L: 0.023 R: 0.014
Arrigoni et al., 2024 [62] Italy	Retrospective	n = 51 (n = 16 COVID-CM; n = 35 COVID-OD) COVID-CM median (IQR) 56 (51–61) years; COVID-OD median (IQR): 40 (31–53) years COVID-CM (m:f) (5: 11), COVID-OD (m:f) (10:25)	n = 14 median (IQR) 62 (45–70) years (m:f) (8:6)	Post-COVID olfactory and cognitive impairment	Median (IQR): 264 (208–313) days	↓	<0.001
Dadsena et al., 2025 [42] Germany	Longitudinal	n = 79 46.43 ± 11.28 years (m:f) (31:48)	n = 21 40.63 ± 14.54 years (m:f) (13:8)	Post-COVID	23 months	↓	<0.05
Besteher et al., 2022 [34] Germany	Cross-sectional	n = 30 47.5 ± 11.5 years (m:f) (13:17)	n = 20 42.95 ± 13.41 years (m:f) (10:10)	Long COVID	8.65 months	↑	L: 0.028 R:0.046
Hafiz et al., 2022 [63] India	Cross-sectional	n = 46 34.63 ± 11.54 years (m:f) (31:15)	n = 30 33.5 ± 9.74 years (m:f) (23:7)	Recovered	2 weeks	↑	<0.05
Tu et al., 2021 [57] China	Prospective	n = 47 51.8 ± 11.3 years (m:f) (14:33)	n = 47 51.8 ± 11.3 years (m:f) (15:32)	Recovered	6 months	↑	<0.05
Lukina et al., 2022 [47] Russia	Cross-sectional Longitudinal Pre- and post-infection vs. controls	n = 34 20–71 years (m:f) (13:21)	n = 30 Comparable to cases in sex and age	Recovered	4–12 months	↑	≤0.05
Lu et al., 2020 [20] China	Prospective	n = 60 44.1 ± 16.0 years (m:f) (34:26)	n = 39 45.9 ± 13.9 years (m:f) (22:17)	Recovered	97.5 ± 8.0 days	↔	L: 0.080 R: 0.463
Zhou et al., 2025 [59] China	Longitudinal	n = 53 (n = 26 SD; n = 27 NSD) SD: 51.5 ± 13.57 years; NSD: 47.33 ± 15.98 Years SD (m:f) (18:8), NSD (m:f) (18:9)	n = 31 49.19 ± 17.51 years (m:f) (22:9)	Post-COVID sleep disturbances	3 months	↔	L: 0.061 R: 0.016
Invernizzi et al., 2024 [45] Italy	Case–control Longitudinal	n = 13 23.76 ± 2.82 years (m:f) (6:7)	n = 27 24.1 ± 2.3 years (m:f) (8:19)	Recovered	1–11 months	↔	0.404
Taskiran-Sag et al., 2023 [56] Turkey	Cross-sectional case–control	n: 20 35.5 ± 9.5 years (m:f) (10:10)	n = 20 36.3 ± 6.7 years (m:f) (9:11)	Recovered	107 days	↔	R:0.465 L: 0.066
Bispo et al., 2022 [65] Brazil	Cross-sectional	n = 56 37.2 ± 9.4 years (m:f) (20:36)	n = 37 40.2 ± 11.8 years (m:f) (15:22)	Post-COVID symptoms	93.3 ± 26.4	↔	>0.120
Hosp et al., 2024 [68] Germany	Cross-sectional	n = 89 median (IQR) 49 (23) years (m:f) (34:55)	n = 46 median (IQR) 44 (31) years (m:f) (23:23)	Post-COVID	254 (209) days median (IQR)	↔	1.000

Abbreviations: m:f, male: female; IQR, interquartile range; CD, cognitive dysfunction; OD, olfactory dysfunction; CM, cognitive impairment; SD, sleep disturbances; NSD, none sleep disturbances; R, right; L, left; GMV, grey matter volume.

### 3.9. Thalamic Volume

More than half of the studies (8 out of 15, 53%; n = 762 patients, n = 268 controls) reported lower thalamic volumes [33,35,40,41,43,53,58,66]. Two studies (13%, n = 54 patients, n = 44 controls) reported higher thalamic volumes in patients with post-COVID syndrome [34,54], and five studies (33%, n = 305 patients, n = 200 controls) reported no volumetric differences [20,59,61,65,68] (Table 6).

**Table 6:** Thalamic volume.

First Author, Year	Study Design	Sample Characteristics		COVID-19 Severity	Time Between COVID-19 and MRI	Brain Volume Results	*p*-Value
		COVID-19	Control				
Qin et al., 2021 [53] China	Prospective	n = 51 (n = 19 mild; n = 32 severe) Mild: 59.4 ± 5.9 years; Severe: 63.2 ± 5.4 years Mild (m:f) (7:12), Severe (m:f) (16:16)	n = 31 60.58 ± 6.42 years (m:f) (18:13)	Recovered	101.21 ± 12.24 days	↓	R: 0.0084 L: 0.0370
Jin et al., 2023 [70] China	Longitudinal (Pre-Post Infection)	n = 21 24.38 ± 2.26 years (m:f) (0:21)	Patients acted as their own controls	Recovered	59.10 ± 10.01 days	↓	NR
Bendella et al., 2023 [33] Germany	Prospective	n = 99 (n = 51 mild; n = 48 severe) Mild: 45.7 ± 12.4 years; Severe: 50.6 ± 12.0 years Mild (m:f) (28:28), Severe (m:f) (25:23)	n = 56 47.0 ± 13.3 years (m:f) (26:25)	Recovered	Mild: 8.7 ± 4.8 months Severe: 10.7 ± 5 months	↓	R: <0.001 L: 0.03
Diez-Cirarda et al., 2025 [40] Spain	Cross-sectional	n = 129 49.35 ± 10.29 years (m:f) (34:95)	n = 36 56.9 ± 13.6 years (m:f) (20:16)	Long COVID	14.79 ± 7.17 months	↓	0.005
C-MORE 2023 [41] UK	Prospective	n = 259 57.0 ± 12.2 years (m:f) (158:101)	n = 52 49.3 ± 13.9 years (m:f) (30:22)	Recovered	Median (IQR): 5 (4.2–6.3) months	↓	<0.05
Capelli et al., 2024 [35] Italy	Retrospective	n = 145 (n = 61 COVID-CD; n = 48 COVID-OD) COVID-CD median [IQR] 57 [50–63] years; COVID-OD median [IQR]: 49 [35–57] years COVID-CD (m:f) (23:38), COVID-OD (m:f) (34:50)	n = 17 Median [IQR] 51 [41–52] years (m:f) (10:7)	COVID-19-related cognitive and olfactory dysfunction	COVID-CD median [IQR]:210 [53–446] days COVID-OD median [IQR]: 237 [180–323] days	↓	<0.0001
Tian et al., 2022 [58] China	Prospective Longitudinal	n = 34 (n = 13 mild; n = 21 severe) Mild: 58.2 ± 5.7; Severe: 62.8 ± 5.3 years Mild (m:f) (6:7), Severe (m:f) (10:11)	n = 31 60.58 ± 6.42 years (m:f) (18:13)	Recovered	302.7 ± 15.6 days	↓	0.0350
González-Rosa et al., 2024 [66] Spain	Cross-sectional Longitudinal	n = 24 45.17 ± 10.66 years (m:f) (9:15)	n = 24 39.67 ± 10.24 years (m:f) (8:16)	Recovered	9 months	↓	<0.05
Rothstein et al., 2023 [54] USA	Retrospective	n = 24 46.9 years (range 22–60 years) (m:f) (5: 19)	Sex and age matched healthy controls	Post- COVID syndrome	85 days	↑	<0.0001
Besteher et al., 2022 [34] Germany	Cross-sectional	n = 30 47.5 ± 11.5 Years (m:f) (13:17)	n = 20 42.95 ± 13.41 years (m:f) (10:10)	Long COVID	8.65 months	↑	0.044
Zhou et al., 2025 [59] China	Longitudinal	n = 53 (n = 26 SD; n = 27 NSD) SD: 51.5 ± 13.57 years; NSD: 47.33 ± 15.98 Years SD (m:f) (18:8), NSD (m:f) (18:9)	n = 31 49.19 ± 17.51 years (m:f) (22:9)	Post- COVID sleep disturbances	3 months	↔	L: 0.081 R: 0.162
Lu et al., 2020 [20] China	Prospective	n = 60 44.1 ± 16.0 years (m:f) (34:26)	n = 39 45.9 ± 13.9 years (m:f) (22:17)	Recovered	97.5 ± 8.0 days	↔	L: 0.544 R: 0.520
Heine et al., 2023 [61] Germany	Cross-sectional	n = 47 43.4 ± 11.9 years (m:f) (8:39)	n = 47 44.5 ± 14.1 years (m:f) (8:39)	Post-COVID fatigue	Median (IQR): 7.5 (6.5–9.2) months	↔	L: 0.09 R: 0.33
Bispo et al., 2022 [65] Brazil	Cross-sectional	n = 56 37.2 ± 9.4 years (m:f) (20:36)	n = 37 40.2 ± 11.8 years (m:f) (15:22)	Post- COVID symptoms	93.3 ± 26.4	↔	>0.120
Hosp et al., 2024 [68] Germany	Cross-sectional	n = 89 median (IQR) 49 (23) years (m:f) (34:55)	n = 46 median (IQR) 44 (31) years (m:f) (23:23)	Post-COVID	254 (209) days median (IQR)	↔	1.000

Abbreviations: m:f, male, female; R, right; L, left; NR, not reported; IQR, interquartile range; CD, cognitive dysfunction; OD, olfactory dysfunction; PCS, post-COVID syndrome; GMV, grey matter volume; SD, sleep disturbances; NSD, none sleep disturbances.

### 3.10. Basal Ganglia Volume

Putamen: Lower volume was the most prevalent finding (8 of 13 studies, 62%; n = 815 patients, n = 284 controls) [33,35,40,41,53,58,61,62]. Higher volume was reported in two studies (15%, n = 76 patients, n = 50 controls) by Besteher et al. [34] and Hafiz et al. [63], and three studies by Lu et al. [20] Bispo et al. [65] and Hosp et al. [68] found no significant difference.

Caudate: Findings were distributed across 10 studies: 4 reported lower volume (40%; n = 584 patients, n = 119 controls) [35,40,41,62], two found significantly larger caudate (20%, n = 64 patients, n = 50 controls) [34,47] and four found no change (40%, n = 252 patients, n = 169 controls) [20,61,65,68].

Pallidum: Results from 10 studies were also mixed: three reported lower volume (30%, n = 629 patients; n = 476 controls) reported lower volumes in patients compared to controls [17,33,40], three others reported higher volume (30%, n = 110 patients, n = 80 controls) [34,47,63], and four did not detect any differences (40%, n = 252 patients, n = 169 controls) [20,61,65,68] (Table 7).

**Table 7:** Basal ganglia volume.

Basal Ganglia Part	First Author, Year Country	Study Design	Sample Characteristics		COVID-19 Severity	Time Between COVID-19 and MRI	Direction	*p*
			COVID-19	Control				
Caudate	Besteher et al., 2022 [34] Germany	Cross-sectional	n = 30 47.5 ± 11.5 years (m:f) (13:17)	n = 20 42.95 ± 13.41 years (m:f) (10:10)	Long COVID	8.65 months	↑	0.046
	Lukina et al., 2022 [47] Russia	Cross-sectional Longitudinal Pre- and post-infection vs. controls	n = 34 20–71 years (m:f) (13:21)	n = 30 comparable to cases in sex and age	Recovered	4–12 months	↑	≤0.05
	Arrigoni et al., 2024 [62] Italy	Retrospective	n = 51 (n = 16 COVID-CM; n = 35 COVID-OD) COVID-CM median (IQR) 56 (51–61) years; COVID-OD median (IQR): 40 (31–53) years COVID-CM (m:f) (5: 11), COVID-OD (m:f) (10:25)	n = 14 median (IQR) 62 (45–70) years (m:f) (8:6)	Post- COVID olfactory and cognitive impairment	Median (IQR): 264 (208–313) days	↓	<0.001
	Diez-Cirarda et al., 2025 [40] Spain	Cross-sectional	n = 129 49.35 ± 10.29 years (m:f) (34:95)	n = 36 56.9 ± 13.6 years (m:f) (20:16)	Long COVID	14.79 ± 7.17 months	↓	L: 0.009 R: 0.003
	Capelli et al., 2024 [35] Italy	Retrospective	n = 145 (n = 61 COVID-CD; n = 48 COVID-OD) COVID-CD median (IQR) 57 (50–63) years; COVID-OD median (IQR) 49 (35–57) years COVID-CD (m:f) (23:38), COVID-OD (m:f) (34:50)	n = 17 Median (IQR) 51 (41–52) years (m:f) (10:7)	COVID-19-related cognitive and olfactory dysfunction	COVID-CD median (IQR) 210 (53–446) days COVID-OD median (IQR) 237 (180–323) days	↓	<0.0001
	C-MORE 2023 [41] UK	Prospective	n = 259 57.0 ± 12.2 years (m:f) (158:10)	n = 52 49.3 ± 13.9 years (m:f) (30:22)	Recovered	Median (IQR): 5 (4.2–6.3) (ml)	↓	L: 0.030 R: 0.025
	Bispo et al., 2022 [65] Brazil	Cross-sectional	n = 56 37.2 ± 9.4 years (m:f) (20:36)	n = 37 40.2 ± 11.8 years (m:f) (15:22)	Post- COVID symptoms	93.3 ± 26.4	↔	>0.120
	Lu et al., 2020 [20] China	Prospective	n = 60 44.1 ± 16.0 years (m:f) (34:26)	n = 39 45.9 ± 13.9 years (m:f) (22:17)	Recovered	97.5 ± 8.0 days	↔	L: 0.296 R: 0.832
	Hosp et al., 2024 [68] Germany	Cross-sectional	n = 89 median (IQR) 49 (23) years (m:f) (34:55)	n = 46 median (IQR) 44 (31) years (m:f) (23:23)	Post-COVID	254 (209) days median (IQR)	↔	1.000
	Heine et al., 2023 [61] Germany	Cross-sectional	n = 47 43.4 ± 11.9 years (m:f) (8:39)	n = 47 44.5 ± 14.1 years (m:f) (8:39)	Post-COVID fatigue	7.5 (6.5–9.2) Months median (IQR)	↔	L: 0.54 R: 0.88
Putamen	Bendella et al., 2023 [33] Germany	Prospective	n = 99 (n = 51 mild; n = 48 severe) Mild: 45.7 ± 12.4 years; Severe: 50.6 ± 12.0 years Mild (m:f) (28:28) Severe (m:f) (25:23)	n = 56 47.0 ± 13.3 years (m:f) (26:25)	Recovered	Mild: 8.7 ± 4.8 months Severe: 10.7 ± 5 months	↓	R: 0.006 L:0.004
	Tian et al., 2022 [58] China	Prospective Longitudinal	n = 34 (n = 13 mild; n = 21 severe) Mild: 58.2 ± 5.7; Severe: 62.8 ± 5.3 years Mild (m:f) (6:7) Severe (m:f) (10:11)	n = 31 60.58 ± 6.42 years (m:f) (18:13)	Recovered	302.7 ± 15.6 days	↓	L: 0.040 R: 0.015
	C-MORE 2023 [41] UK	Prospective	n = 259 57.0 ± 12.2 years (m:f) (158:101)	n = 52 49.3 ± 13.9 years (m:f) (30:22)	Recovered	Median (IQR): 5 months (4.2–6.3)	↓	<0.001
	Capelli et al., 2024 [35] Italy	Retrospective	n = 145 (n = 61 COVID-CD; n = 48 COVID-OD) COVID-CD median (IQR) 57 (50–63) years; COVID-OD median (IQR) 49 (35–57) years COVID-CD (m:f) (23:38), COVID-OD (m:f) (34:50)	n = 17 Median (IQR) 51 (41–52) years (m:f) (10:7)	COVID-19-related cognitive and olfactory dysfunction	COVID-CD median (IQR) 210 (53–446) days COVID-OD median (IQR) 237 (180–323) days	↓	<0.0001
	Diez-Cirarda et al., 2025 [40] Spain	Cross-sectional	n = 129 49.35 ± 10.29 years (m:f) (34:95)	n = 36 56.9 ± 13.6 years (m:f) (20:16)	Long COVID	14.79 ± 7.17 months	↓	0.009
	Qin et al., 2021 [53] China	Prospective	n = 51 (n = 19 mild; n = 32 severe) Mild: 59.4 ±5.9 years; Severe: 63.2 ± 5.4 years Mild (m:f) (7:12), Severe (m:f) (16:16)	n = 31 60.58 ± 6.42 years (m:f) (18:13)	Recovered	101.21 ± 12.24 days	↓	0.0202
	Heine et al., 2023 [61] Germany	Cross-sectional	n = 47 43.4 ± 11.9 years (m:f) (8:39)	n = 47 44.5 ± 14.1 years (m:f) (8:39)	Post-COVID fatigue	Median (IQR): 7.5 (6.5–9.2) months	↓	L: 0.049 R: 0.18
	Arrigoni et al., 2024 [62] Italy	Retrospective	n = 51 (n = 16 COVID-CM; n = 35 COVID-OD) COVID-CM median (IQR) 56 (51–61) years; COVID-OD median (IQR): 40 (31–53) years COVID-CM (m:f) (5: 11), COVID-OD (m:f) (10:25)	n = 14 median (IQR) 62 (45–70) years (m:f) (8:6)	Post- COVID olfactory and cognitive impairment	Median (IQR): 264 (208–313) days	↓	R COVID-CM vs. Control: 0.009 R COVID-OD vs. Control: 0.002
	Besteher et al., 2022 [34] Germany	Cross-sectional	n = 30 47.5 ± 11.5 years (m:f) (13:17)	n = 20 42.95 ± 13.41 years (m:f) (10:10)	Long COVID	8.65 months	↑	0.028
	Hafiz et al., 2022 [63] India	Cross-sectional	n = 46 34.63 ± 11.54 years (m:f) (31:15)	n = 30 33.5 ± 9.74 years (m:f) (23:7)	Recovered	2 weeks	↑	<0.05
	Bispo et al., 2022 [65] Brazil	Cross-sectional	n = 56 37.2 ± 9.4 years (m:f) (20:36)	n = 37 40.2 ± 11.8 years (m:f) (15:22)	Post-COVID symptoms	93.3 ± 26.4	↔	> 0.120
	Hosp et al., 2024 [68] Germany	Cross-sectional	n = 89 median (IQR) 49 (23) years (m:f) (34:55)	n = 46 median (IQR) 44 (31) years (m:f) (23:23)	Post- COVID	254 (209) days median (IQR)	↔	1.000
	Lu et al., 2020 [20] China	Prospective	n = 60 44.1 ± 16.0 years (m:f) (34:26)	n = 39 45.9 ± 13.9 years (m:f) (22:17)	Recovered	97.5 ± 8.0 days	↔	L: 0.712 R: 0.972
Pallidum	Douaud et al., 2022 [17] UK	Longitudinal Pre- and post-infection vs. controls	n = 401 62.1 ± 6.7 years (m:f) (172:229)	n = 384 63.3 ± 7.1 years (m:f) (164:220)	Recovered	141 ± 79 days (second scan).	↓	<0.05
	Bendella et al., 2023 [33] Germany	Prospective	n = 99 (n = 51 mild; n = 48 severe) Mild: 45.7 ± 12.4 years; Severe: 50.6 ± 12.0 years Mild (m:f) (28:28), Severe (m:f) (25:23)	n = 56 47.0 ± 13.3 years (m:f) (26:25)	Recovered	Mild: 8.7 ± 4.8 months Severe: 10.7 ± 5 months	↓	R: 0.014 L: 0.005
	Diez-Cirarda et al., 2025 [40] Spain	Cross-sectional	n = 129 49.35 ± 10.29 years (m:f) (34:95)	n = 36 56.9 ± 13.6 years (m:f) (20:16)	Long COVID	14.79± 7.17 months	↓	0.005
	Besteher et al., 2022 [34] Germany	Cross-sectional	n = 30 47.5 ± 11.5 years (m:f) (13:17)	n = 20 42.95 ± 13.41 years (m:f) (10:10)	Long COVID	8.65 months	↑	L: 0.028 R: 0.046
	Lukina et al., 2022 [47] Russia	Cross-sectional Longitudinal Pre- and post-infection vs. controls	n = 34 20–71 years (m:f) (13:21)	n = 30 Comparable to cases in sex and age	Recovered	4–12 months	↑	≤0.05
	Hafiz et al., 2022 [63] India	Cross-sectional	n = 46 34.63 ± 11.54 years (m:f) (31:15)	n = 30 33.5 ± 9.74 years (m:f) (23:7)	Recovered	2 weeks	↑	<0.05
	Bispo et al., 2022 [65] Brazil	Cross-sectional	n = 56 37.2 ± 9.4 years (m:f) (20:36)	n = 37 40.2 ± 11.8 years (m:f) (15:22)	Post- COVID symptoms	93.3 ± 26.4	↔	>0.120
	Hosp et al., 2024 [68] Germany	Cross-sectional	n = 89 median (IQR) 49 (23) years (m:f) (34:55)	n = 46 median (IQR) 44 (31) years (m:f) (23:23)	Post-COVID	254 (209) days median (IQR)	↔	1.000
	Lu et al., 2020 [20] China	Prospective	n = 60 44.1 ± 16.0 years (m:f) (34:26)	n = 39 45.9 ± 13.9 years (m:f) (22:17)	Recovered	97.5 ± 8.0 days	↔	L: 0.972 R: 0.920
	Heine et al., 2023 [61] Germany	Cross-sectional	n = 47 43.4 ± 11.9 years (m:f) (8:39)	n = 47 44.5 ± 14.1 years (m:f) (8:39)	Post-COVID fatigue	7.5 (6.5–9.2) median (IQR) months	↔	L: 0.85 R: 0.94

Abbreviations: m:f, male: female; GMV, grey matter volume; CM, cognitive impairment; OD, olfactory dysfunction; CD, cognitive dysfunction; IQR, interquartile range; R, right; L, left.

### 3.11. Nucleus Accumbens Volume

Results for the nucleus accumbens were equally divided, with 4 of 8 studies (50%; n = 254 patients, n = 82 controls) reporting lower volume [35,58,60,62], and the other four (50%; n = 252 patients, n = 169 controls) reporting no significant change [20,61,65,68] (Table 8).

**Table 8:** Nucleus accumbens volume.

First Author, Year Country	Study Design	Sample Characteristics		COVID-19 Severity	Time Between COVID-19 and MRI	Direction	*p*
		COVID-19	Control				
Tian et al., 2022 [58] China	Prospective Longitudinal	n = 34 (n = 13 mild; n = 21 severe) Mild: 58.2 ± 5.7; Severe: 62.8 ± 5.3 years Mild (m:f) (6:7) Severe (m:f): (10:11)	n = 31 60.58 ± 6.42 years (m:f) (18:13)	Recovered	302.7 ± 15.6 days	↓	0.0480
Arrigoni et al., 2024 [62] Italy	Retrospective	n = 51 (n = 16 COVID-CM; n = 35 COVID-OD) COVID-CM median (IQR) 56 (51–61) years; COVID-OD median (IQR): 40 (31–53) years COVID-CM (m:f) (5:11), COVID-OD (m:f) (10:25)	n = 14 median (IQR) 62 (45–70) years (m:f) (8:6)	Post- COVID olfactory and cognitive impairment	Median (IQR): 264 (208–313) days	↓	<0.001
Trufanov et al., 2025 [60] Russia	Cross-sectional Case–control	n = 24 49.16 ± 10.65 years (m:f) (12:11)	n = 20 42.84 ± 8.93 years (m:f) (6:12)	Post-COVID-syndrome	4–6 months	↓	L: 0.023 R: 0.014
Capelli et al., 2024 [35] Italy	Retrospective	n = 145 (n = 61 COVID-CD; n = 48 COVID-OD) COVID-CD median (IQR) 57 (50–63) years; COVID-OD median (IQR) 49 (35–57) years COVID-CD (m:f) (23:38), COVID-OD (m:f) (34:50)	n = 17 Median (IQR) 51 (41–52) years (m:f) (10:7)	COVID-19-related cognitive and olfactory dysfunction	COVID-CD median (IQR) 210 (53–446) days COVID-OD median (IQR) 237 (180–323) days	↓	<0.0001
Lu et al., 2020 [20] China	Prospective	n = 60 44.1 ± 16.0 years (m:f) (34:26)	n = 39 45.9 ± 13.9 years (m:f) (22:17)	Recovered	97.5 ± 8.0 days	↔	L: 0.053 R: 0.321
Heine et al., 2023 [61] Germany	Cross-sectional	n = 47 43.4 ± 11.9 years (m:f) (8:39)	n = 47 44.5 ± 14.1 years (m:f) (8:39)	Post-COVID fatigue	7.5 (6.5–9.2) Months median (IQR)	↔	L: 0.26 R: 0.47
Bispo et al., 2022 [65] Brazil	Cross-sectional	n = 56 37.2 ± 9.4 years (m:f) (20:36)	n = 37 40.2 ± 11.8 years (m:f) (15:22)	Post- COVID symptoms	93.3 ± 26.4	↔	>0.120
Hosp et al., 2024 [68] Germany	Cross-sectional	n = 89 49 (23) years median (IQR) (m:f) (34:55)	n = 46 median (IQR) 44 (31) years (m:f) (23:23)	Post- COVID	254 (209) days median (IQR)	↔	1.000

Abbreviations: m:f, male: female; NAC, nucleus accumbens; CM, cognitive impairment; CD, cognitive dysfunction; OD, olfactory dysfunction; IQR, interquartile range; TIV, total intracranial volume.

### 3.12. Cerebellar Volume

A strong majority of studies (80%; n = 1068 patients, n = 608 controls) reported lower cerebellar volumes [17,18,34,35,36,41,46,47], while Lu et al. [20] and Hosp et al. [68] (20%, n = 149 patients, n = 85 controls) did not find a statistically significant difference (Table 9).

**Table 9:** Cerebellar volume.

First Author, Year	Study Design	Sample Characteristics		COVID-19 Severity	Time Between COVID-19 and MRI	Direction	*p*
		COVID-19	Control				
Douaud et al., 2022 [17] UK	Longitudinal Pre- and post-infection vs. controls	n = 401 62.1 ± 6.7 years (m:f) (172:229)	n = 384 63.3 ± 7.1 years (m:f) (164:220)	Recovered	141 ± 79 days (second scan).	↓	<0.05
Besteher et al., 2022 [34] Germany	Cross-sectional	n = 30 47.5 ± 11.5 years (m:f) (13:17)	n = 20 42.95 ± 13.41 years (m:f) (10:10)	Long COVID	8.65 months	↓	0.024
Cataldo et al., 2024 [36] Argentina	Cross-sectional	n = 109 48.4 ± 8.0 years (m:f) (30:79)	n = 28 45.2 ± 9.9 years (m:f) (9:19)	Long COVID	2 years	↓	0.03
Kamasak et al., 2023 [46] Turkey	Cross-sectional	n = 50 38.10 ± 5.85 years (m:f) (25:25)	n = 50 38.78 ± 6.16 years (m:f) (25:25)	Recovered	17 days	↓	<0.001
Capelli et al., 2024 [35] Italy	Retrospective	n = 145 (n = 61 COVID-CD; n = 48 COVID-OD) COVID-CD median (IQR) 57 (50–63) years; COVID-OD median (IQR) 49 (35–57) years COVID-CD (m:f) (23:38), COVID-OD (m:f) (34:50)	n = 17 Median (IQR) 51 (41–52) years (m:f) (10:7)	COVID-19-related cognitive and olfactory dysfunction	COVID-CD median (IQR) 210 (53–446) days COVID-OD median (IQR) 237 (180–323) days	↓	Cerebellar vermal lobules I–V: <0.0001 R cerebellum exterior: 0.035
C-MORE 2023 [41] UK	Prospective	n = 259 57.0 ± 12.2 years (m:f) (158:101)	n = 52 49.3 ± 13.9 years (m:f) (30:22)	Recovered	Median [IQR]: 5 [4.2–6.3] months	↓	Cerbellum Vermis ix: 0.053
Du et al., 2022 [18] China	Prospective Longitudinal	n = 22 1-year; n = 18 2-years 1-year: 54.2 ± 10; 2-years: 53.5 ± 10 years 1-year (m:f) (11:11) 2-year (m:f) (9:9)	n = 27 50.8 ± 11.5 years (m:f) (7:20)	Recovered	1-year: 342.8 ± 15.1 days 2-years: 731.8 ± 13.2 days	↓	0.001
Lukina et al., 2022 [47] Russia	Cross-sectional Longitudinal Pre- and post-infection vs. controls	n = 34 20–71 years (m:f) (13:21)	n = 30 comparable to cases in sex and age	Recovered	4–12 months	↓	R: ≤0.01 L: ≤0.05
Lu et al., 2020 [20] China	Prospective	n = 60 44.1 ± 16.0 years (m:f) (34:26)	n = 39 45.9 ± 13.9 years (m:f) (22:17)	Recovered	97.5 ± 8.0 days	↔	L: 0.981 R: 0.920
Hosp et al., 2024 [68] Germany	Cross-sectional	n = 89 49 (23) years median (IQR) (m:f) (34:55)	n = 46 44 (31) years median (IQR) (m:f) (23:23)	Post-COVID	254 (209) days median (IQR)	↔	1.000

Abbreviations: m:f, male: female; GMV, grey matter volume; IQR, interquartile range; CD, cognitive dysfunction; OD, olfactory dysfunction.

## 4. Discussion

We summarized findings from 41 studies with 4629 participants (including 2895 cases), reporting post-COVID-19 volumetric brain changes across different regions. The hippocampus, amygdala, thalamus, basal ganglia, nucleus accumbens and the cerebellum noted significant changes, with reduction being the most apparent direction of change. This pattern was particularly noticeable in severe COVID-19 cohorts [33,41,53,58]. The total white matter volume appeared most resistant to change, with no study reporting statistically significant differences between COVID-19 cases and controls [33,36,38,41,46,49]. Overall, substantial variability in results has been evident.

The most reliable conclusions regarding volumetric reductions in patients compared to controls can be drawn for the hippocampus and cerebellum, supported by a substantial proportion of Good quality studies (62% and 63%, respectively). Evidence for the amygdala is also strong, with 57% of studies rated as Good. In contrast, findings for subcortical structures are more mixed. The thalamus and caudate show moderate support, with 50% of studies rated as Good, while the putamen (38%) and nucleus accumbens (25%) are less certain, relying mainly on lower-quality studies. Although the pallidum shows a relatively high proportion of Good quality studies (67%), this conclusion is based on very few reports and should therefore be considered tentative (Supplementary Table S4).

Despite the discrepancy between studies, this review’s main finding is that SARS-CoV-2 infection could be associated with long-term and brain-region-specific structural alterations. The persistence of observed volume reduction for years after acute illness in the hippocampus or amygdala may not be surprising, given that post-COVID patients report long-term memory deficits [71] and anxiety symptoms [72]. Similarly, cerebellar changes and the subtle gait abnormalities [73,74] reported in these patients could be related. However, volumetric changes could be attributed to a variety of pathological processes. For instance, increased volume could be related to an inflammatory response, which is more likely to take place during the early post-COVID period. Hafiz et al., whose COVID-19 patients had a short duration from acute illness to imaging (2 weeks), showed larger grey matter volumes in patients compared to controls [63].

The brain region-specific structural alteration could be a manifestation of different routes of viral CNS invasion. Several possible routes of invasion have been proposed. First, the nasal cavity is considered the most common route of infection due to the high expression of ACE2 receptors in this region [15]. Infection of the nasal epithelium may allow the virus to spread to the olfactory bulb via the olfactory nerves [15,75]. Second, the high viral load in the respiratory tracts may lead to tissue damage, enabling the virus to hematogenously spread and infect any organ expressing ACE2 receptors, including the brain [15]. Hematogenous spread occurs when the virus is present in the blood stream and infects endothelial cells of the blood–brain barrier (BBB) or infects leucocytes. Infected leucocytes cross to the brain carrying the virus [76] and cause activation of microglia and release of proinflammatory cytokines (e.g., TNF-alpha, IL-1-beta, IL-6) by both neurons and astrocytes [77,78]. Third, ocular transmission is a possible route for brain infection [79,80]. Viral transport through the optic nerve may enable the virus to reach the occipital cortex [81,82,83]. Other mechanisms of how SARS-CoV-2 affects the brain include inflammatory cytokine storm, microvasculitis, and hypoxia, all of which can increase BBB permeability and cause neuronal damage [84,85]. However, more studies are needed to elucidate the effect of viral invasion route on affected brain regions.

Although several biological pathways have been proposed to explain these volumetric alterations—including neuroinflammation, hypoxia, microvascular injury, it is important to note that these remain theoretical within the context of the studies included in this review. None of the 41 studies incorporated serum or CSF biomarkers (e.g., cytokines, NfL, GFAP, and ferritin) or linked volumetric findings to biological indicators of inflammation, hypoxia, or neuronal injury. Similarly, only a small number of studies provided longitudinal imaging [17,18,42,45,47,49,55,58,59], which limits our ability to determine whether these changes reflect transient inflammatory swelling, progressive neuronal loss, or a combination of processes. Therefore, while the proposed mechanisms are biologically plausible, they should be interpreted as hypotheses rather than confirmed explanatory pathways. Future research integrating structural MRI with biomarker data and repeated follow-up scans will be essential to clarify the underlying pathology and to distinguish reversible changes from permanent tissue loss.

Despite the variable findings of lower, higher, or unchanged volumes across different brain regions, the consistent no change in total white matter volume in the six included studies seems intriguing, especially with reports of white matter abnormalities, using other MRI modalities.

Diffusion MRI methods were also reported. Huang et al. noted persistent white matter microstructural abnormalities using Diffusion tensor imaging (DTI) and the neurite orientation dispersion and density index (NODDI) in recovered COVID patients (n = 17) after two years and revisited healthy controls (n = 13) [86]. In a prospective study, Rau et al. compared whole-brain white matter Diffusion Microstructure Imaging (DMI) parameters between subacute COVID-19 patients (n = 20) and healthy controls (n = 35). They found widespread volume shifts into the free water fraction (V-CSF), affecting white matter fibers connecting widespread cortical regions in all cerebral lobes [87]. These results imply that COVID-related white matter affection could be related to microscale abnormalities and impaired white matter integrity rather than a macroscale volumetric change.

Volumetric reductions, on the other hand, could suggest a reduced number of cells or disrupted tissue cell layer arrangement. Cell death through different mechanisms, such as apoptosis, ferroptosis, pyroptosis, and necrosis, has been reported following neurotropic viral infections [88]. Apoptosis was reported in SARS-CoV-2 infected human microglia cell line (HCM3) [89], and activation of necrotic pathways was reported in mouse neuronal culture [90]. The pyroptosis signature was detected in some critical care COVID-19 patients [91]. We reviewed the potential occurrence of ferroptosis and its relation to neuropsychiatric manifestations of COVID-19 [92,93]. Notably, the neuropathology of SARS-CoV-2 could be attributed not only to the full virus but also to the SARS-CoV-2 spike protein, which can cross the blood–brain barrier and induce pathological changes [94]. However, further studies are needed to assess the relation between cell death and brain volumetric changes and the effect of SARS-CoV-2 spike protein on brain volumetric measures.

Another prominent finding that leaves us with more questions than answers is the substantial variability in the results across studies. This is clearly seen in regions like the hippocampus, amygdala and thalamus where the number of studies reporting decreases, increases, or no change is nearly equal. This can be potentially due to (1) differences in hardware (scanner, field strength, and coil type), software (imaging protocol parameters), and data processing [95,96,97,98], (2) comorbid medical and psychiatric conditions such as obesity, BMI [99], diabetes [100], hypertension [101] depression and anxiety [102], (3) COVID-19 related variables such as severity of illness, time interval from acute illness to brain imaging, and whether the patient developed post-COVID syndrome and, (4) age and sex differences [103,104]. In a direct examination of these demographic factors, a study by Haider et al. used linear regression analysis and found that both age and sex were significant predictors of total grey matter volume. Their model showed that grey matter volume decreased by an estimated 2.44 mL per year with increasing age. Furthermore, female participants exhibited significantly lower grey matter volumes compared to males, with an average difference of 61.13 mL [67]. These findings highlight the prominent role of demographic factors, such as age and sex, in explaining volumetric differences, emphasizing the importance of adjusting for these variables when examining post-COVID effects on brain structure. As expected, studies used a wide range of scanners, imaging protocols, and software (Supplementary Table S5) on patients and controls with different comorbidities. In fact, several studies did not provide data on comorbidities [18,35,40,42,43,45,54,57,59,61,62,63,66,67,69] (Table 10).

Another source of heterogeneity across studies was the inconsistency in adjusting volumetric measures for total intracranial volume (TIV), a standard normalization step in structural MRI to account for inter-individual differences in head size. Several studies applied TIV correction when reporting grey matter and white matter [17,20,34,35,36,37,38,39,40,42,43,45,46,48,49,51,54,57,59,60,63,64,65,66,69,70], whereas others did not [18,33,41,44,47,50,52,53,55,56,58,61,62,67,68]. Since volumetric differences related to COVID-19 may be subtle, a lack of TIV correction can either inflate group differences (by attributing head-size variability to disease effects) or obscure true effects (by increasing unexplained variance). This methodological inconsistency likely contributed to some of the conflicting findings across studies and highlights the need for standardized analytic pipelines in future work.

Our findings align with prior systematic reviews examining post-COVID structural brain alterations. Consistent with Nelson et al. [26], we observed that gray matter reductions were most apparent among patients with more severe illness or hospitalization. Similarly, Alhazmi et al. [28] reported significant subcortical gray matter volume loss in the bilateral thalamus, caudate, and putamen, reinforcing the vulnerability of deep gray matter nuclei to post-infectious effects. In contrast, a recent multimodal voxel-based morphometry meta-analysis [27] described decreased gray matter volume in the anterior cingulate and medial prefrontal cortices and left cerebellum alongside increases in the bilateral amygdala and hippocampus. This divergence likely arises from several factors, including differences in analytic frameworks (meta-analytic versus narrative approaches), methodological variability, and cohort characteristics such as illness severity, confounding variables, and the timing of post-infection imaging. Collectively, this heterogeneity highlights the need for standardized imaging protocols, longitudinal study designs, and rigorous control of known confounders in future research.

To our knowledge, this is the first comprehensive literature review to summarize volumetric brain changes in SARS-CoV-2-infected individuals compared to non-infected controls, regardless of symptom status or time since infection. It also identifies key trends and patterns, offering a strong foundation for future research into the long-term neurological sequelae of COVID-19.

## 5. Limitations

A few limitations of the included studies should be acknowledged. The first limitation is that 46% of the included studies were cross-sectional in design [34,36,37,38,39,40,46,47,50,51,52,56,60,61,63,64,65,68,69], which makes it challenging to identify causality. Although some longitudinal data were available, the small number and variable follow-up intervals meant that cross-sectional and longitudinal findings had to be summarized together, limiting conclusions about temporal trajectories of brain alterations. The second limitation is that no antibody testing was performed on COVID-negative controls to ensure that they had never been infected, and since asymptomatic SARS-CoV-2 infection is associated with volumetric changes [17], this might have attenuated some of the observed volumetric differences between patients and controls. A third limitation is that few studies reported their participants’ vaccination status [42,43,45,50,55,62], and only six [37,41,43,48,65,66] specified the coronavirus variant—two factors that may significantly alter brain morphology. The fourth limitation is the varied MRI software and methods used, which might explain some of the heterogeneity in the results, and preclude a formal meta-analysis, requiring a narrative synthesis instead. Relatedly, several clinical confounders such as hospitalization, steroid use, and comorbidities were reported inconsistently and therefore could not be evaluated as moderators. A fifth limitation of our review is that it pooled all COVID-19 cases, including those with post-COVID syndrome or post-COVID neurological disease, or those who were fully recovered. Taken together, it is difficult to confidently attribute specific volumetric changes to the COVID-19 virus alone, as they may also arise from post-COVID symptoms (e.g., cognitive impairment). Sixth, although the search strategy was comprehensive, we did not formally assess publication bias, and the under-representation of null findings cannot be excluded. Lastly, our review mainly focused on MRI studies that assessed structural alterations separately. Future research should take into account multimodal MRI studies to provide complementary information on both brain structure and function, which will help improve our understanding of the mechanisms contributing to volumetric changes post-infection.

As shown by Huang and Rau, DTI, tractography and advanced diffusion MRI models methods such as NODDI and the standard model of diffusion (SMT) have shown promise in detecting microstructural differences in white matter regions affected by COVID-19 by assigning signal fractions to specific compartments of a simplified neuronal environment, i.e., myelinated axons, extracellular space, and free water. While these methods are valuable to characterize white matter tissue, they are not optimized for investigating grey matter. To further investigate the microstructural characteristics of gray matter, where significant volumetric reductions were observed, implementation of diffusion MRI models specifically designed to account for features more prevalent in gray matter, such as changes in soma volumes and higher prevenance of microglia [105,106] may offer valuable insights into grey matter microstructural characteristics. Although implementing these models presents certain challenges, including longer acquisition times, their specificity to gray matter makes them promising tools for advancing our understanding of the underlying microstructural alterations caused by COVID-19 infection.

## 6. Conclusions

The current literature, despite inconsistencies, suggests long-term brain volumetric changes after exposure to SARS-CoV-2 infection, specifically in patients who survived severe infections but also in asymptomatic cases. Given that hundreds of millions were exposed to this infection, identifying a true negative-control group is challenging, and the potential confounding effect of such non-specific changes on imaging studies for other disorders should be considered.

We have to conclude that marked incongruence exists in the literature regarding brain volume changes after COVID-19 infection. This highlights the need to investigate not only whether clinical characteristics, MRI techniques, and COVID-19 severity contribute to heterogeneity in structural brain volumes but also whether further stratification of COVID-19 patients into post-COVID syndrome or post-COVID neurological disease cohorts may help reduce these discrepancies and reveal clearer trends.

## Figures and Tables

**Table 10:** Comorbidities and hospitalization rates.

First Author, Year	Comorbidities		Hospitalization Rate (%)
	COVID-19	Control	
Bendella et al., 2023 [33]	No relevant comorbidities	No relevant comorbidities	49%
Besteher et al., 2022 [34]	No relevant comorbidities	No relevant comorbidities	13.33%
Capelli et al., 2024 [35]	NR	NR	NR
Cataldo et al., 2024 [36]	Hypertension: 30.4% Diabetes: 5.5% Asthma: 7.3% High cholesterol: 25.7% Heart attack: 0.9% Angina pectoris: 0.9% Embolism or thrombosis: 1.8%	Hypertension: 25.9% Diabetes: 3.7% Asthma: 0% High cholesterol: 29.6% Heart attack: 0% Angina pectoris: 0% Embolism or thrombosis: 0%	16.5%
Cattarinussi et al., 2022 [37]	No relevant comorbidities	No relevant comorbidities	28%
Cecchetti et al., 2022 [38]	Hypertension: 41.7% Dyslipidemia: 22.2% Diabetes mellitus: 11.1% Obesity: 5.6%	Hypertension: 22.2% Dyslipidemia: 36.1% Diabetes mellitus: 2.8% Obesity: 11.1%	86.1%
Díez-Cirarda et al., (2023) [39]	Hypertension: 23.8% Diabetes: 10.7% Dyslipidemia: 26.2%	Hypertension: 12.12% Diabetes: 3.03% Dyslipidemia: 12.12%	33.33%
Diez-Cirarda et al., 2025 [40]	Hypertension: 25.6% Diabetes: 10.1% Dyslipidemia: 24.0%	NR	27.9%
C-MORE., 2023 [41]	Diabetes: 21% High cholesterol: 18% Hypertension: 50% Respiratory comorbidity: 32% Cardiac comorbidity: 16% Neurological comorbidity: 4% Liver disease: 5% Kidney disease: 5%	Diabetes: 13% High cholesterol: 13% Hypertension: 29% Respiratory comorbidity: 15% Cardiac comorbidity: 4% Neurological comorbidity: 2% Liver disease: 0% Kidney disease: 2%	100%
Dadsena et al., 2025 [42]	Cardiovascular risk factors: 36.7% Neurological comorbidities: 19% Psychiatric comorbidities: 8.9%	NR	19%
Du et al., 2023 [43]	Hypertension: 14.8% Type 2 diabetes: 9.8%	NR	NR
Du et al., 2023 [18]	NR	NR	100%
Griffanti et al., 2021 [44]	Hypertension: 54.5% Diabetes: 18.6%	Hypertension: 25% Diabetes: 8.7%	100%
Invernizzi et al., 2024 [45]	NR	NR	NR
Jin et al., 2024 [70]	NR	NR	0%
Kamasak et al., 2023 [46]	No relevant comorbidities	No relevant comorbidities	0%
Lu et al., 2020 [20]	Hypertension: 21.7% Diabetes: 10.0%	Hypertension: 41.03% Diabetes: 2.56%	100%
Lukina et al., 2022 [47]	No relevant comorbidities	No relevant comorbidities	0%
Muccioli et al., 2023 [48]	No relevant comorbidities	No relevant comorbidities	8.7%
Niu et al., 2025 [49]	Comorbidity (any): 6.0% Hypertension: 1.8% Diabetes: 0.0% Hyperlipidemia: 0.9% Respiratory disease: 0.6%	Comorbidity (any): 3.8% Hypertension: 0.0% Diabetes: 1.3% Hyperlipidemia: 0.0% Respiratory disease: 0.0%	NR
Okrzeja et al., 2024 [50]	Hypertension: 43.5% Diabetes: 17.4% Hyperlipidemia: 8.7%	No relevant comorbidities	100%
Perlaki et al., 2024 [51]	No relevant comorbidities	No relevant comorbidities	0%
Pelizzari et al., 2022 [52]	The two groups were matched for hypertension (Fisher’s exact test, *p* = 0.233), hyperlipidemia (Fisher’s exact test, *p* = 0.488). Diabetes: 0%		0%
Qin et al., 2021 [53]	Hypertension Mild: 16% Severe: 53% Diabetes Mild: 11% Severe: 22% Coronary Heart Disease Mild: 5% Severe:9%	Hypertension: 35% Diabetes: 6%	100%
Rothstein et al., 2023 [54]	Depression or anxiety: 29.1% Diabetes: 12.5% Seizure disorder: 4.1%	NR	0%
Syunyakov et al., 2022 [55]	Any chronic disease: 91.7% Diabetes: 20.8% Hypertension: 75% Ischemic heart disease: 29.2% Myocardial infarction history: 4.2% Oncology history: 29.2% Obesity: 16.7% Any affective disorder history: 16.7% Any anxiety disorder history: 12.5% OCD: 0.0%	Any chronic disease: 90.7% Diabetes: 13.3% Hypertension: 65.5% Ischemic heart disease: 28.3% Myocardial infarction history: 3.3% Oncology history: 15.0% Obesity: 21.1% Any affective disorder history: 12.8% Any anxiety disorder history: 2.8% OCD: 0.6%	16.7%
Taskiran-Sag et al., 2023 [56]	No relevant comorbidities	No relevant comorbidities	0%
Tu et al., 2021 [57]	Hypertension: 23%, Diabetes:6%	NR	100%
Tian et al., 2022 [58]	Hypertension: Mild 15%, severe 52% Diabetes: Mild 15%, severe 29% Coronary heart disease: Mild 8%, severe 14%	Hypertension: 35% Diabetes: 6% Coronary heart disease: 3%	100%
Zhou et al., 2025 [59]	NR	NR	NR
Trufanov et al., 2025 [60]	No relevant comorbidities	No relevant comorbidities	NR
Heine et al., 2023 [61]	Hypertension: 11% Hypothyroidism: 9% Depression: 6.4% Anxiety: 6.4%	NR	13%
Arrigoni (2024) [62]	NR	NR	NR
Hafiz et al., 2022 [63]	NR	NR	100%
Douaud et al., 2022 [17]	Diabetes: 4.5%	Diabetes: 4.2%	3.74%
Gupta et al., 2024 [64]	Anxiety/ Depression: 10% Hypertension: 14%	Anxiety/ Depression: 10% Hypertension: 20%	100%
Bispo et al., 2022 [65]	Hypertension: 8.9% Diabetes mellitus: 8.9% Obesity: 1.8% Asthma/COPD: 3.6% Mood disorder: 7.1%	Hypertension: 8.1% Diabetes mellitus: 8.1% Obesity: 8.1% Asthma/COPD: 5.4% Mood disorder: 5.4%	5%
González-Rosa et al., 2024 [66]	-	-	0%
Haider et al., 2025 [67]	-	-	73%
Hosp et al., 2024 [68]	Hypertension: 21% Asthma: 9% Diabetes: 6% CHD: 3% CKF: 1%	Hypertension: 0% Asthma: 2% Diabetes: 3% CHD: 2% CKF: 0%	15%
Thapaliya et al., 2025 [69]	-	-	-

Abbreviations: NR, not reported; OCD, obsessive–compulsive disorder; CHD, coronary heart disease; CKF, chronic kidney failure.

## Data Availability

No data was used for the research described in the article.

## Supplementary Materials

The following supporting information can be downloaded at: https://www.mdpi.com/article/10.3390/brainsci15121255/s1.
