# Supplementary material for: Brain Volumetric Changes Post-COVID-19: A Systematic Review
Source: Brain Sci. 2025 Nov 22;15(12):1255. doi: 10.3390/brainsci15121255 (PMC12730699; doi:10.3390/brainsci15121255)
Supplement: Supplementary file 1 [file brainsci-15-01255-s001.zip › brainsci-3972978-supplementary.pdf]

Supplementary Appendix

This supplementary material for the article entitled:

Brain Volumetric Changes Post-COVID-19: A Systematic Narratie Review

Table of Contents

Supplementary Tables 2

Supplementary Table S1: PRISMA 2020 Main Checklist 2

Supplementary Table S2: PRIMSA Abstract Checklist 5

Supplementary Table S3: Search strategy 6

Supplementary Table S4: NIH NHLBI Quality Assessment for Observational Cohort and Cross-Sectional Studies for all included studies. 7

Supplemetary Table S5: MRI acquisition and data processing methods 10

Supplementary Figures 13

Supplementary Figure S1: NIH NHLBI Quality Assessment for Observational Cohort and Cross-Sectional Studies Evaluation for Included Studies 13

Supplementary Tables

Supplementary Table S1: PRISMA 2020 Main Checklist

Topic	No.	Item	Location where item is reported
**TITLE**			
**Title**	1	Identify the report as a systematic review.	1
**ABSTRACT**			
**Abstract**	2	See the PRISMA 2020 for Abstracts checklist	
**INTRODUCTION**			
**Rationale**	3	Describe the rationale for the review in the context of existing knowledge.	4
**Objectives**	4	Provide an explicit statement of the objective(s) or question(s) the review addresses.	4
**METHODS**			
**Eligibility criteria**	5	Specify the inclusion and exclusion criteria for the review and how studies were grouped for the syntheses.	4&5
**Information sources**	6	Specify all databases, registers, websites, organisations, reference lists and other sources searched or consulted to identify studies. Specify the date when each source was last searched or consulted.	5
**Search strategy**	7	Present the full search strategies for all databases, registers and websites, including any filters and limits used.	5 & Supplementary Table 3
**Selection process**	8	Specify the methods used to decide whether a study met the inclusion criteria of the review, including how many reviewers screened each record and each report retrieved, whether they worked independently, and if applicable, details of automation tools used in the process.	5

Topic	No.	Item	Location where item is reported
**Data collection process**	9	Specify the methods used to collect data from reports, including how many reviewers collected data from each report, whether they worked independently, any processes for obtaining or confirming data from study investigators, and if applicable, details of automation tools used in the process.	5
**Data items**	10a	List and define all outcomes for which data were sought. Specify whether all results that were compatible with each outcome domain in each study were sought (e.g. for all measures, time points, analyses), and if not, the methods used to decide which results to collect.	5
	10b	List and define all other variables for which data were sought (e.g. participant and intervention characteristics, funding sources). Describe any assumptions made about any missing or unclear information.	5
**Study risk of bias assessment**	11	Specify the methods used to assess risk of bias in the included studies, including details of the tool(s) used, how many reviewers assessed each study and whether they worked independently, and if applicable, details of automation tools used in the process.	6
**Effect measures**	12	Specify for each outcome the effect measure(s) (e.g. risk ratio, mean difference) used in the synthesis or presentation of results.	-
**Synthesis methods**	13a	Describe the processes used to decide which studies were eligible for each synthesis (e.g. tabulating the study intervention characteristics and comparing against the planned groups for each synthesis (item 5)).	-
	13b	Describe any methods required to prepare the data for presentation or synthesis, such as handling of missing summary statistics, or data conversions.	-
	13c	Describe any methods used to tabulate or visually display results of individual studies and syntheses.	-
	13d	Describe any methods used to synthesize results and provide a rationale for the choice(s). If meta-analysis was performed, describe the model(s), method(s) to identify the presence and extent of statistical heterogeneity, and software package(s) used.	-
	13e	Describe any methods used to explore possible causes of heterogeneity among study results (e.g. subgroup analysis, meta-regression).	-
	13f	Describe any sensitivity analyses conducted to assess robustness of the synthesized results.	-
**Reporting bias assessment**	14	Describe any methods used to assess risk of bias due to missing results in a synthesis (arising from reporting biases).	-
**Certainty assessment**	15	Describe any methods used to assess certainty (or confidence) in the body of evidence for an outcome.	-
**RESULTS**			

Topic	No.	Item	Location where item is reported
**Study selection**	16a	Describe the results of the search and selection process, from the number of records identified in the search to the number of studies included in the review, ideally using a flow diagram.	6
	16b	Cite studies that might appear to meet the inclusion criteria, but which were excluded, and explain why they were excluded.	6
**Study characteristics**	17	Cite each included study and present its characteristics.	6 & Table 2-10
**Risk of bias in studies**	18	Present assessments of risk of bias for each included study.	7 & Supplementary Table 4 & Supplementary Fig 1
**Results of individual studies**	19	For all outcomes, present, for each study: (a) summary statistics for each group (where appropriate) and (b) an effect estimate and its precision (e.g. confidence/credible interval), ideally using structured tables or plots.	-
**Results of syntheses**	20a	For each synthesis, briefly summarise the characteristics and risk of bias among contributing studies.	-
	20b	Present results of all statistical syntheses conducted. If meta-analysis was done, present for each the summary estimate and its precision (e.g. confidence/credible interval) and measures of statistical heterogeneity. If comparing groups, describe the direction of the effect.	-
	20c	Present results of all investigations of possible causes of heterogeneity among study results.	-
	20d	Present results of all sensitivity analyses conducted to assess the robustness of the synthesized results.	-
**Reporting biases**	21	Present assessments of risk of bias due to missing results (arising from reporting biases) for each synthesis assessed.	-
**Certainty of evidence**	22	Present assessments of certainty (or confidence) in the body of evidence for each outcome assessed.	-
**DISCUSSION**			
**Discussion**	23a	Provide a general interpretation of the results in the context of other evidence.	10
	23b	Discuss any limitations of the evidence included in the review.	13
	23c	Discuss any limitations of the review processes used.	-
	23d	Discuss implications of the results for practice, policy, and future research.	14
**OTHER INFORMATION**			
**Registration and protocol**	24a	Provide registration information for the review, including register name and registration number, or state that the review was not registered.	4

Topic	No.	Item	Location where item is reported
	24b	Indicate where the review protocol can be accessed, or state that a protocol was not prepared.	4
	24c	Describe and explain any amendments to information provided at registration or in the protocol.	-
**Support**	25	Describe sources of financial or non-financial support for the review, and the role of the funders or sponsors in the review.	15
**Competing interests**	26	Declare any competing interests of review authors.	15
**Availability of data, code and other materials**	27	Report which of the following are publicly available and where they can be found: template data collection forms; data extracted from included studies; data used for all analyses; analytic code; any other materials used in the review.	15

Supplementary Table S2: PRIMSA Abstract Checklist

Topic	No.	Item	Reported?
**TITLE**			
**Title**	1	Identify the report as a systematic review.	Yes
**BACKGROUND**			
**Objectives**	2	Provide an explicit statement of the main objective(s) or question(s) the review addresses.	Yes
**METHODS**			
**Eligibility criteria**	3	Specify the inclusion and exclusion criteria for the review.	Yes
**Information sources**	4	Specify the information sources (e.g. databases, registers) used to identify studies and the date when each was last searched.	Yes
**Risk of bias**	5	Specify the methods used to assess risk of bias in the included studies.	No
**Synthesis of results**	6	Specify the methods used to present and synthesize results.	-
**RESULTS**			
**Included studies**	7	Give the total number of included studies and participants and summarise relevant characteristics of studies.	Yes
**Synthesis of results**	8	Present results for main outcomes, preferably indicating the number of included studies and participants for each. If meta-analysis was done, report the summary estimate and confidence/credible interval. If comparing groups, indicate the direction of the effect (i.e. which group is favoured).	-

Topic	No.	Item	Reported?
**DISCUSSION**			
**Limitations of evidence**	9	Provide a brief summary of the limitations of the evidence included in the review (e.g. study risk of bias, inconsistency and imprecision).	No
**Interpretation**	10	Provide a general interpretation of the results and important implications.	Yes
**OTHER**			
**Funding**	11	Specify the primary source of funding for the review.	No
**Registration**	12	Provide the register name and registration number.	No

Supplementary Table S3: Search strategy

Data Base	Search Query	N
**PubMed**<br><br>date: 09/4/2025	((COVID-19) OR (SARS-CoV-2) OR (COVID-19)) AND (Brain) AND ((Magnetic Resonance Imaging) OR (MRI))<br><br>((COVID 19) OR (Corona virus) OR (SARS-CoV-2)) AND ((Nucleus Accumbens) OR (Caudate) OR (cerebell*)) AND ((MRI) OR (Magnetic resonance imaging))	1699
**Scopus**<br>Filter: title/abstract/ keywords<br><br>date: 09/4/2025	((COVID-19) OR (SARS-CoV-2) OR (COVID-19)) AND (Brain) AND ((Magnetic Resonance Imaging) OR (MRI))<br><br>((COVID 19) OR (Corona virus) OR (SARS-CoV-2)) AND ((Nucleus Accumbens) OR (Caudate) OR (cerebell*)) AND ((MRI) OR (Magnetic resonance imaging))	3590

**Web Of Science**<br><br>date: 09/4/2025	((COVID-19) OR (SARS-CoV-2) OR (COVID-19)) AND (Brain) AND ((Magnetic Resonance Imaging) OR (MRI))<br><br>((COVID 19) OR (Corona virus) OR (SARS-CoV-2)) AND ((Nucleus Accumbens) OR (Caudate) OR (cerebell*)) AND ((MRI) OR (Magnetic resonance imaging))	1167
**Google Scholar**<br><br>date: 09/04/2025	allintitle: Covid Brain MRI	29
**Embase**<br><br>date: 09/09/2025	((COVID-19) OR (SARS-CoV-2) OR (COVID-19)) AND (Brain) AND ((Magnetic Resonance Imaging) OR (MRI))<br><br>((COVID 19) OR (Corona virus) OR (SARS-CoV-2)) AND ((Nucleus Accumbens) OR (Caudate) OR (cerebell*)) AND ((MRI) OR (Magnetic resonance imaging))	4757

Supplementary Table S4: NIH NHLBI Quality Assessment for Observational Cohort and Cross-Sectional Studies for all included studies.

**Study ID**	**1**	**2**	**3**	**4**	**5**	**6**	**7**	**8**	**9**	**10**	**11**	**12**	**13**	**14**	**Rating**
Besteher et al., 2022	Y	Y	NR	Y	N	Y	Y	Y	Y	NA	Y	NA	NA	Y	Good
Perlaki et al., 2023	Y	Y	Y	Y	N	Y	Y	NA	N	NA	Y	N	NA	Y	Good
C-MORE 2023	Y	Y	N	NR	Y	Y	Y	Y	Y	NA	N	NA	NA	Y	Fair

Du et al., 2022	Y	Y	N	NR	N	Y	Y	N	Y	NR	Y	NA	Y	Y	Good
Cataldo et al., 2024	Y	Y	NR	Y	Y	Y	Y	N	Y	NA	Y	NA	NA	Y	Good
Bendella et al., 2023	Y	N	Y	Y	Y	Y	Y	Y	N	NA	Y	NA	NA	N	Poor
Tian et al., 2022	Y	Y	NR	NR	N	Y	Y	Y	N	NR	Y	NA	N	Y	Fair
Tu et al., 2021	Y	N	NR	N	Y	Y	Y	N	Y	NR	Y	NA	NA	Y	Poor
Qin et al., 2021	Y	Y	NR	NR	N	Y	Y	Y	N	NA	Y	NA	NA	N	Poor
Lu et al., 2020	Y	Y	NR	NR	N	Y	Y	Y	N	NA	Y	NA	NA	Y	Fair
Douaud et al., 2022	Y	Y	NR	Y	N	Y	Y	Y	Y	NA	Y	NA	NA	Y	Good
Cattarinussi et al., 2022	Y	Y	Y	Y	N	Y	Y	N	Y	NA	Y	NA	NA	Y	Good
Cecchetti et al., 2022	Y	Y	N	Y	N	Y	Y	N	Y	NR	N	N	Y	Y	Fair
Kamasak et al., 2023	Y	Y	NR	Y	N	Y	Y	N	N	NA	Y	NA	NA	Y	Fair
Invernizzi et al., 2024	Y	Y	N	Y	Y	Y	Y	N	Y	NR	Y	NR	N	Y	Good
Muccioli et al., 2023	Y	Y	NR	Y	Y	Y	Y	N	Y	NR	Y	NR	N	Y	Good
Zhou et al., 2024	Y	Y	NR	Y	Y	Y	Y	Y	Y	NR	Y	NR	N	Y	Good
Syunyakov et al., 2022	Y	Y	N	Y	Y	Y	Y	N	N	NR	Y	NR	N	Y	Fair
Taskiran-Sag et al., 2023	Y	Y	NR	Y	Y	Y	Y	N	Y	NA	Y	NA	NA	Y	Good
Rothstein et al., 2023	Y	Y	NR	Y	N	Y	Y	N	Y	NR	Y	NR	Y	Y	Good
Díez-Cirarda	Y	Y	Y	Y	N	Y	Y	N	Y	NA	Y	NA	NA	Y	Good

et al., 2023															
Diez-Cirarda et al., 2025	Y	Y	NR	Y	N	Y	Y	N	Y	NA	Y	NA	NA	Y	Good
Capelli et al., 2024	Y	Y	N	Y	N	Y	Y	Y	Y	NR	Y	NR	Y	Y	Good
Lukina et al., 2022	Y	N	N	Y	N	Y	Y	N	N	NA	Y	NA	NA	Y	Poor
Niu et al., 2025	Y	Y	Y	Y	Y	Y	Y	N	N	NR	Y	NR	N	Y	Good
Pelizzari et al., 2022	Y	N	NR	Y	Y	Y	Y	Y	Y	NA	Y	NA	NA	Y	Fair
Trufanov et al., 2025	Y	N	NR	Y	N	Y	Y	N	Y	NA	Y	NA	NA	N	Poor
Heine et al., 2023	Y	Y	NR	Y	N	Y	Y	Y	Y	NA	Y	NA	NA	Y	Good
Arrigoni et al., 2024	Y	N	Y	Y	N	Y	Y	N	Y	NR	Y	NR	Y	Y	Fair
Hafiz et al., 2020	Y	Y	N	N	Y	Y	Y	N	Y	NA	Y	NA	NA	Y	Fair
Griffanti et al., 2021	Y	Y	NR	Y	Y	Y	Y	N	Y	NA	Y	NR	Y	Y	Good
Jin et al., 2024	Y	Y	NR	Y	Y	Y	Y	N	Y	NR	Y	NR	Y	Y	Good
Dadsena et al., 2025	Y	Y	NR	Y	N	Y	Y	N	Y	NR	Y	NR	Y	Y	Good
Cecchetti et al., 2022	Y	Y	N	Y	Y	Y	Y	N	Y	NA	Y	NA	NA	Y	Good
Du et al., 2023	Y	Y	Y	Y	N	Y	Y	N	Y	NR	Y	NR	N	Y	Fair
Bispo et al., 2022	Y	Y	Y	Y	Y	Y	Y	N	Y	NA	Y	NA	Y	Y	Good
González-Rosa et al., 2024	Y	Y	Y	Y	Y	Y	Y	N	Y	NR	Y	NR	Y	Y	Good
Gupta et al., 2024	Y	Y	Y	Y	Y	Y	Y	N	N	NA	Y	NA	Y	Y	Good

Hosp et al., 2024	Y	Y	Y	Y	Y	Y	Y	Y	Y	NA	Y	NA	Y	Y	Good
Haider et al., 2025	Y	Y	Y	Y	Y	Y	Y	N	Y	NR	Y	NR	Y	Y	Good
Thapaliya et al., 2025	Y	Y	Y	Y	N	Y	Y	N	Y	NA	Y	NA	Y	Y	Fair

Y – Yes; N – No; NR – Not Reported; NA – Not Applicable

Supplemetary Table S5: MRI acquisition and data processing methods

Study (Year)	Scanner (Field Strength, Model)	Sequence	TR (ms)	TE (ms)	FOV (mm)	Processing Software
Bendella et al., 2023	3T, Philips Achieva TX	3D T1w MPRAGE	7.3	3.9	256 × 256	mdbrain (v4.4.1)
Besteher et al., 2022	3T, Siemens Tim Trio	3D T1w MPRAGE	2400	2.22	256	CAT12
Capelli et al., 2024	3T, General Electric	T1w MEMP	600 (COVID), 9 (Control)	9 (COVID), 480 (Control)	250 × 250	CAT12
Cataldo et al., 2024	3T, Siemens Magneton Prisma	T1w MPRAGE	2000	2.98	-	FSL SIENAX (v6.0.7.7), FreeSurfer (v7.4)
Cattarinussi et al., 2022	3T, Philips Ingenia	3D T1w MPRAGE	6676	3	240	DPABI, SPM12
Cecchetti et al., 2022	3T, Philips Intera	3D T1w FFE	7	3.2	256 × 240	FSL SIENAX (v5.0.9)
Díez-Cirarda et al., 2023	3T, GE Signa Architect	3D T1w MPRAGE	7.7	3.1	256	FreeSurfer (v7.2.0)
Diez-Cirarda et al., 2025	3T, GE Signa Architect	3D T1w MPRAGE	2450	3.1	256	SPM12/DARTEL, FreeSurfer (v7.2.0), MARSbar

C-MORE., 2023	3T Siemens/Philips	T1w MPRAGE	Siemens TRouter/TRinner: 2000/1.95, Philips TRouter/TRinner: 2000/7	Siemens: 2.22, Philips: 3.2	-	FSL, FreeSurfer
Dadsena et al., 2025	3T Siemens Prisma/Prisma Fit	T1w MPRAGE	Prisma: 2.4, Prisma Fit: 2.5	Prisma: 2.36, Prisma Fit: 2.64	-	CAT12
Du et al., 2023	3T Siemens Skyra/uMR790	3D T1w MPRAGE	2000	Skyra: 2.26, uMR790: 3.1	256 × 256	FreeSurfer (v6.0)
Du et al., 2022	3T, MAGNETOM Skyra	3D T1w MPRAGE	2000	2.26	256 mm × 256 mm	FSL-VBM
Griffanti et al., 2021	3T, Siemens Prisma	T1w MPRAGE	2000	-	-	FSL
Invernizzi et al.,2024	3T, Siemens Skyra	3D T1w MPRAGE	2400	2.06	-	FreeSurfer (v7.1.1)
Jin et al., 2024	3T, GE Discovery MR750	3D T1WI-BRAVO	8.2	3.2	-	DPABI
Kamasak et al., 2023	1.5T, GE SIGNA Explorer	3D T1w MPRAGE	1900	2.67	250	CAT12
Lu et al., 2020	3T, Siemens Skyra	3D T1w MPRAGE	1900	2.84	280 × 280	CAT12
Lukina et al., 2022	1.5T, Toshiba Excelart Vantage XGV	3D-MPRAGE-IP	12	5	256	FreeSurfer (Ubuntu 16.04.1)
Muccioli et al.,2023	3T, Siemens Skyra	3D T1w MPRAGE	2300	2.98	256	FreeSurfer (v6.0)
Niu et al., 2025	Multi-center 3T (GE/Siemens)	3D T1w	Varies (8.5–1.9)	Varies (3.2–2.4)	-	CAT12
Okrzeja	3T, Siemens	3D T1w	700	11	235	-

et al., 2024	Biograph mMR	SPACE FS				
Perlaki et al., 2024	3T, Siemens PrismaFit	3D T1w MPRAGE	2530	3.37	256 × 256	FreeSurfer (v6.0)
Pelizzari et al., 2022	3T, Siemens PRISMA	3D MPRAGE	2300	3.1	-	FSL
Qin el al., 2021	3T, GE Discovery MR750	3D T1w BRAVO	7.1	2.7	240 × 280	Machine-learning–based volBrain pipeline
Rothstein et al., 2023	3T, Siemens Skyra	3D T1w MPRAGE	2300	2.98	-	NeuroQuant® (v2.3)
Syunyakov et al., 2022	1.5T, Toshia	3D T1w MPRAGE	12	5	256	FreeSurfer (v6.0)
Taskiran-Sag et al., 2023	3T, Philips Ingenia	-	-	-	250 × 250	-
Tu et al., 2021	3T GE scanner	T1 weighted fast-spoiled gradient echo sequence	8.16	3.18	256	CAT12
Tian et al., 2022	3T, GE Discovery MR750	3D T1w MPRAGE	-	-	-	ANTs
Zhou et al., 2024	3T, Siemens Vida/Verio	-	Vida: 1900, Verio: 2000	Vida: 2.22, Verio: 2.02	-	FreeSurfer (v7.2)
Trufanov et al., 2025	3T, Siemens Vida	3D T1w MPRAGE	9.8	4.6	256 × 256	FreeSurfer (v7.3)
Heine et al., 2023	3T, Siemens PRISMA	3D T1w MPRAGE	1900	2.22	-	FSL FIRST
Arrigoni et al., 2024	3T, GE Discovery MR750w	-	600 (COVID), 7 (Control)	9 (COVID), 3 (Control	-	FreeSurfer (v7.3.0)

				)		
Hafiz et al., 2020	3T GE scanner	3D T1WI-BRAVO	-	-	256	SPM12
Douaud et al., 2022	3T	T1	-	-		FreeSurfer
Gupta et al., 2024	3T, Philips Ingenia scanner	MPRAGE	-	-	-	CAT12
Bispo et al., 2022	3T, Philips Achieva	3D T1w	2300	-	208×240×256	FreeSurfer (v7.1)
González-Rosa et al., 2024	1.5 T	3D T1w MPRAGE	7.27	3.32	-	CAT12
Haider et al., 2025	3T, Siemens Vida	3D T1w MPRAGE	-	-	-	AIRC
Hosp et al., 2024	3T MAGNETOM Prisma, Siemens	3D T1w MPRAGE	2500	2.82	-	FreeSurfer (v6.0)
Thapaliya et al., 2025	7 T whole-body MRI research scanner, Siemens	T1-MP2RAGE	4300 ms	2.45 ms	-	FreeSurfer (V 7.4.1)

Abbreviations: TR (ms), repetition time (milliseconds); TE (ms), echo time (milliseconds); FOV (mm), field of view (millimeter); 3D, 3 dimensional; MPRAGE, magnetization-prepared rapid acquisition gradient echo; CAT12, Computational Anatomy Toolbox 12; MEMP, multi echo multi planar; FSL, FMRIB Software Library; DAPI, Data Processing & Analysis for Brain Imaging; SPM12, Statistical Parametrical Mapping 12; FFE, fast field echo; VBM, voxel based morphometry; ANTs, Advanced Normalization Tools; AIRC, AI-Rad Companion.

Supplementary Figures

Supplementary Figure S1: NIH NHLBI Quality Assessment for Observational Cohort and Cross-Sectional Studies Evaluation for Included Studies

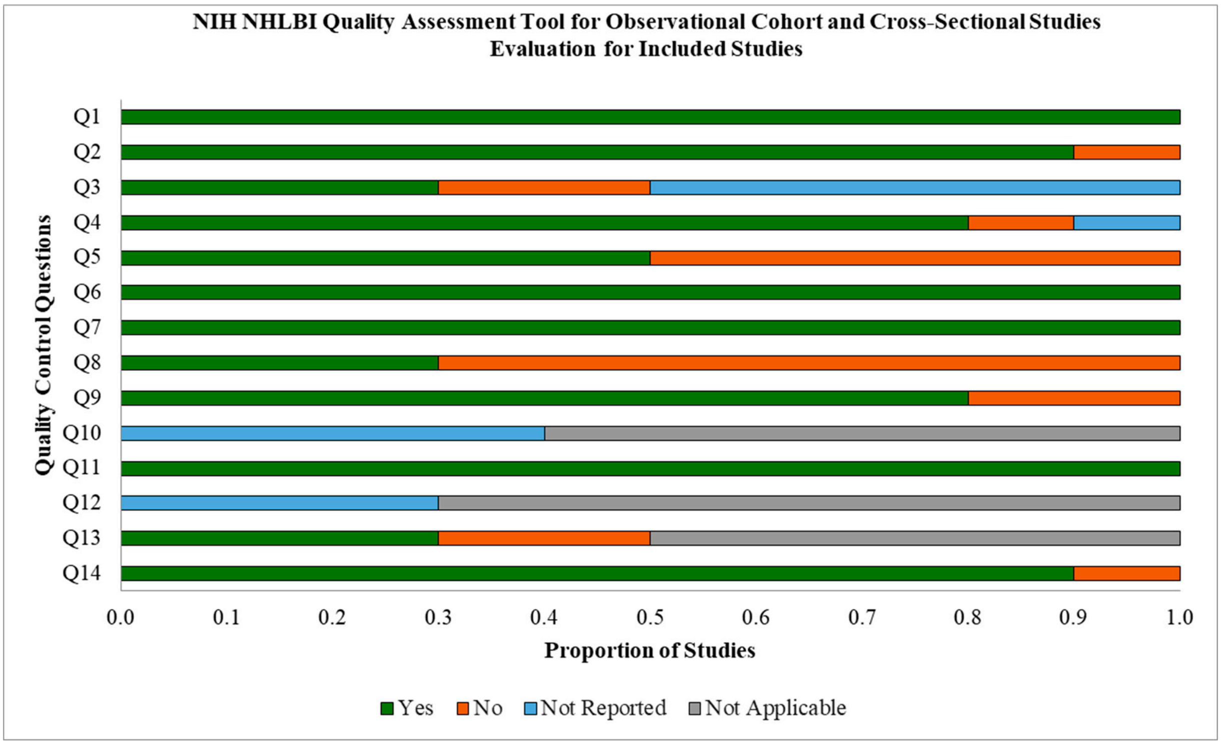

NIH NHLBI Quality Assessment Tool for Observational Cohort and Cross-Sectional Studies
Evaluation for Included Studies
Quality Control Questions
Q1
Q2
Q3
Q4
Q5
Q6
Q7
Q8
Q9
Q10
Q11
Q12
Q13
Q14
0.0
0.1
0.2
0.3
0.4
0.5
0.6
0.7
0.8
0.9
1.0
Proportion of Studies
Yes
No
Not Reported
Not Applicable
